# Proximal Tubule-to-Proximal Tubule Efferocytosis: Gusb Prevents Efferocytosis Failure and Polyphagolysosomal Overload in CAZ-AVI-Induced Acute Kidney Injury

**DOI:** 10.3390/ijms27209045

**Published:** 2026-10-12

**Authors:** Kazuhiro Hasegawa, Yusuke Sakamaki, Masanori Tamaki, Sumiyo Yamaguchi, Ikuko Shimizu, Takahiro Kida, Shinji Miyakami, Miho Tada, Makoto Otsuka, Masanori Minato, Shu Wakino

**Affiliations:** 1 Department of Nephrology, Tokushima University Graduate School of Biomedical Sciences, 3-18-15 Kuramoto-cho, Tokushima 770-8503, Japan; 2 Department of Nephrology and Endocrinology, National Defense Medical College, Tokorozawa 359-8513, Japan

**Keywords:** proximal tubule efferocytosis, drug-induced acute kidney injury, gusb, phagolysosomal dysfunction, polyphagolysosomes, oxidative stress, apoptosis, efferocytic metabolism

## Abstract

Efferocytosis is essential for maintaining epithelial homeostasis, yet whether proximal tubules (PTs) can engulf other PTs and how this process contributes to acute kidney injury (AKI) remain unknown. Ceftazidime–avibactam (CAZ-AVI)-induced AKI and proximal tubule-specific Gusb conditional knockout (CKO) mice both exhibit phagolysosomal dysfunction, suggesting a shared mechanism. We investigated whether (i) CAZ-AVI triggers PT-to-PT efferocytosis, (ii) this leads to polyphagolysosomal failure, and (iii) Gusb overexpression protects against these abnormalities. Wild-type, Gusb-CKO, and Gusb-transgenic mice were treated with CAZ-AVI. Kidney injury was assessed by histology, TUNEL staining, immunostaining for Odc1 and 4-HNE, and transmission electron microscopy. PT-intrinsic transcriptional responses were quantified by real-time PCR for Ccl2, C3, Lcn2, Slc7a13, Acsm3, and Aqp8. Ultrastructural features were evaluated to distinguish efferocytosis from phagolysosomal failure. CAZ-AVI markedly reduced Odc1 in PTs and induced apoptosis localized almost exclusively to PTs, indicating PT-to-PT efferocytosis. CAZ-AVI strongly induced Ccl2, C3, and Lcn2, while suppressing Slc7a13, Acsm3, and Aqp8, reflecting inflammatory recruitment, complement-mediated opsonization, oxidative stress, and metabolic collapse. Electron microscopy revealed numerous polyphagolysosomes containing dense bodies, membrane fragments, small vesicles, and mitochondrial remnants with preserved cristae, without nuclear structures or double-membrane phagosomes, consistent with phagolysosomal failure. Importantly, Gusb-CKO mice phenocopied CAZ-AVI injury, showing Odc1 loss, robust 4-HNE accumulation, PT-restricted apoptosis, and abundant polyphagolysosomes even without drug exposure, demonstrating that Gusb deficiency is sufficient to drive PT-to-PT efferocytosis failure. In contrast, Gusb-overexpressing mice preserved Odc1, prevented apoptosis, normalized all six PT-intrinsic genes, and lacked polyphagolysosomes. CAZ-AVI induces PT-to-PT efferocytosis leading to phagolysosomal overload and oxidative failure. Gusb deficiency reproduces this phenotype, whereas Gusb overexpression preserves PT phagolysosomal homeostasis and prevents AKI progression.

## 1. Introduction

Ceftazidime–avibactam (CAZ-AVI) has rapidly become a therapeutic cornerstone for severe infections caused by carbapenem-resistant bacteria belonging to the order Enterobacterales and other multidrug-resistant Gram-negative organisms, particularly in the recipients of solid organ transplants [1,2]. However, drug-induced acute kidney injury (AKI) has emerged as a major treatment limitation in this population [3,4]. Reportedly, AKI incidence during CAZ-AVI therapy has reached 50% in liver transplant recipients [5,6,7]; additionally, those receiving kidney transplants [8,9] are vulnerable due to donor-derived injury, ischemia–reperfusion stress, and calcineurin inhibitor-induced nephrotoxicity [10]. Despite the increasing clinical use of CAZ-AVI, the mechanisms underlying its nephrotoxicity remain poorly understood.

GUSB is a lysosomal β-glucuronidase responsible for the degradation of glucuronic acid-containing substrates, including glycosaminoglycans. Structural studies [11,12] have shown that human GUSB forms a homotetramer and contains defined active-site motifs and lysosomal-targeting features essential for its catalytic and trafficking functions [13,14].

**Figure 1:**
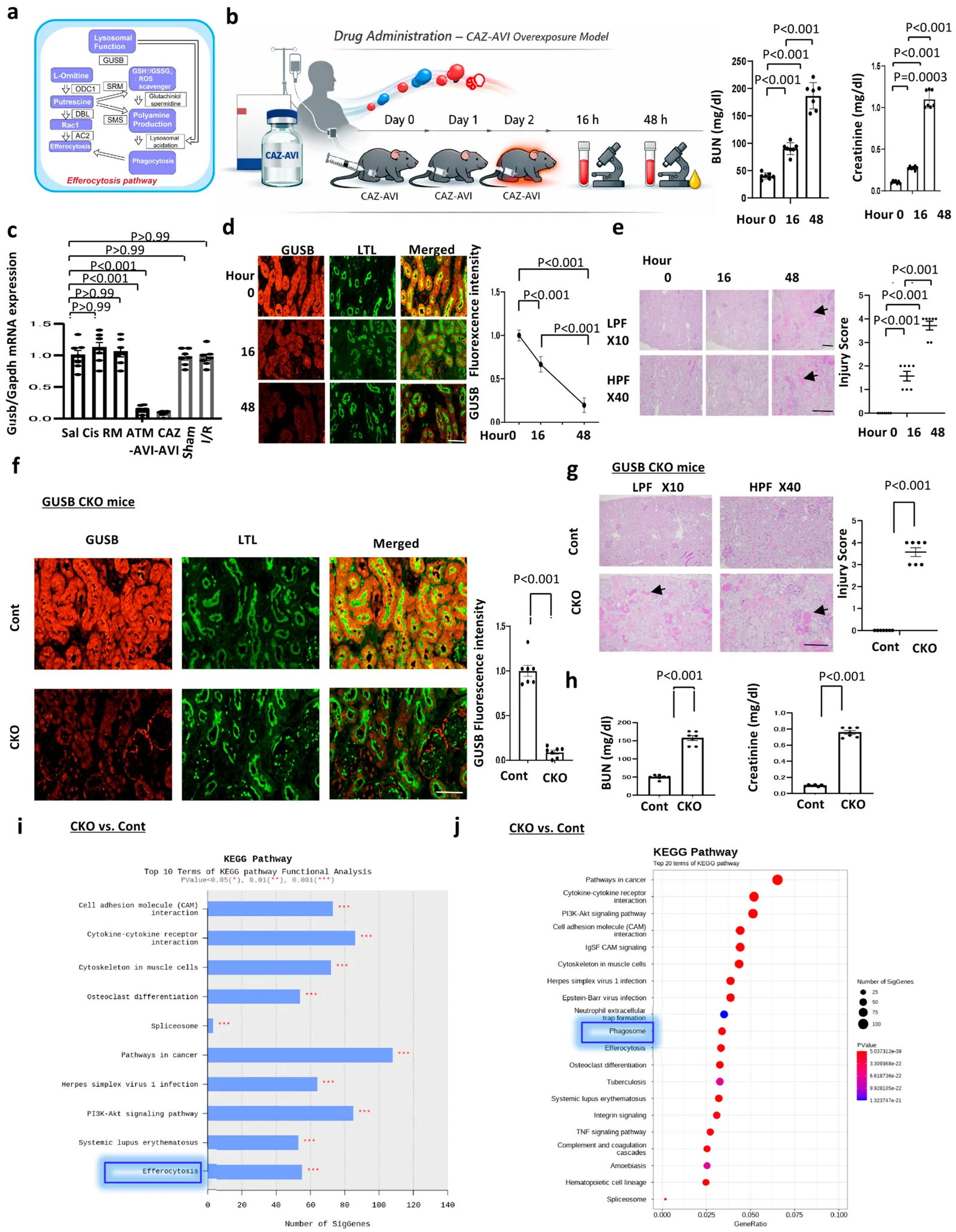
**Generation and characterization of proximal tubule-specific Gusb conditional knockout (CKO) mice and CAZ-AVI-induced AKI model.** (**a**) Schematic representation of the efferocytosis and phagocytosis pathways associated with GUSB-mediated lysosomal function. (**b**) Experimental timeline of the CAZ-AVI overexposure model. Eight-week-old C57BL/6 mice received subcutaneous CAZ-AVI (1250 mg/kg/day) for 3 consecutive days. Serum BUN (left) and creatinine (right) were measured at 0, 16, and 48 h after the final injection. (**c**) Quantitative real-time PCR-based quantification of renal *Gusb* mRNA levels in five AKI models: cisplatin (Cis), rhabdomyolysis (RM), ATM-AVI, CAZ-AVI, and ischemia–reperfusion (I/R). Controls were subjected to sham surgery (Sham) or saline injection (Sal). (**d**) Representative immunofluorescence images of kidney cryosections from CAZ-AVI-treated mice showing GUSB (red) and lotus tetragonolobus lectin (LTL; green) co-labeling of PTs at 0, 16, and 48 h. Quantification of GUSB fluorescence is shown on the right. (**e**) Periodic acid–Schiff (PAS) staining of kidney sections at 0, 16, and 48 h after CAZ-AVI exposure. Arrows indicate lysed tubules. Tubular injury scores are presented on the right. (**f**) Immunofluorescence staining of kidneys from 8-week-old control (Cont) and Gusb-CKO mice (GUSB, red; LTL, green). Quantification of GUSB fluorescence is shown on the right. (**g**) Representative PAS staining of kidney sections from Cont and CKO mice. Arrows point to lysed tubules. Tubular injury scores are presented on the right. (**h**) Serum BUN (left) and creatinine (right) levels in Cont and CKO mice. (**i**) Gene Ontology (GO)-based functional classification of differentially expressed genes (Cont vs. CKO) highlights the enrichment of efferocytosis-related pathways. (**j**) Kyoto Encyclopedia of Genes and Genomes (KEGG) enrichment of differentially expressed genes (*x*-axis: gene ratio; *y*-axis: KEGG terms). Circle size represents the gene count; color indicates the adjusted *p*-value. Phagocytosis-related pathways were enriched. “E” indicates powers of ten. All quantitative data in panels (**b**–**h**) were obtained from *n* = 7 mice per group. Data are presented as the means ± SEM. Statistical analyses were conducted using one-way ANOVA with Bonferroni correction (**b**–**e**) or two-tailed Student’s *t*-test (**f**–**h**). Scale bars: 100 µm (immunofluorescence) and 50 µm (light microscopy). Exact *p*-values are indicated.

Next, we examined the expression of established injury markers during the preliminary analyses of our CAZ-AVI-induced AKI model (Figure 1a,b). As expected, LCN2 and HAVCR1 were induced prominently; however, compared to previous reports, GUSB expression was markedly suppressed in the proximal tubules (PTs) (Figure 1c,d). Such an unexpected downregulation suggested that GUSB is not merely an injury marker but may instead represent a critical determinant of PT homeostasis, whose loss increases susceptibility to drug-induced stress.

These observations led us to hypothesize that GUSB deficiency exacerbates CAZ-AVI-induced AKI by impairing phagolysosomal degradation and overwhelming the capacity of tubular efferocytosis. To test this theory, we generated a PT-specific conditional *Gusb* knockout (*Gusb* CKO) and Gusb transgenic (Gusb Tg) mice (Table 1 and Table 2) and evaluated the impact of Gusb loss or overexpression on CAZ-AVI-induced tubular injury, mitochondrial dysfunction, and efferocytosis dynamics.

Specifically, we now summarize that efferocytosis is an essential mechanism for clearing apoptotic cells and maintaining tissue homeostasis, with established roles in inflammation resolution and tissue repair [15,16,17]. In the kidney, macrophage-mediated efferocytosis has been shown to facilitate recovery from acute injury and regulate neutrophil turnover [17]. We also note that proximal tubule cells possess a highly specialized endolysosomal system that supports protein handling and membrane trafficking, and that disruption of this system contributes to tubular dysfunction and AKI pathogenesis [18,19,20,21]. These additions provide the necessary mechanistic context for understanding the efferocytosis-related phenotypes described in our study.

Transcriptomic analyses of kidneys from deceased donors have identified a set of injury-related genes, including lipocalin-2 (LCN2), hepatitis A virus cellular receptor 1 (HAVCR1/KIM-1), Toll-like receptor 2 (TLR2), interleukin-18 (IL-18), high-mobility group box 1 (HMGB1), caspase-3 (CASP3), and, notably, β-glucuronidase (GUSB); these are upregulated after brain death and are correlated with post-transplant renal outcomes [22,23]. Although these findings suggest that GUSB may be associated with tubular injury, its functional role in proximal tubular biology is yet to be elucidated.

## 2. Results

### 2.1. CAZ-AVI-Induced AKI Is Characterized by Proximal Tubular Gusb Downregulation and Efferocytosis Pathway Activation

As outlined in the latter part of the Introduction, GUSB plays a central role in lysosomal maturation and in the coordination of efferocytosis-related pathways. Building on this framework, we next examined how GUSB expression and tubular injury responses are altered in our CAZ-AVI-induced AKI model.

GUSB is a lysosomal β-glucuronidase that contributes to lysosomal maturation and degradative capacity (Figure 1a), consistent with its established catalytic and trafficking functions described in structural studies [11,12,13,14]. The proximal tubule endolysosomal system plays a central role in protein handling and membrane trafficking, and its disruption has been linked to tubular dysfunction and AKI pathogenesis [18,19,20]. Furthermore, efferocytosis is essential for apoptotic cell clearance and inflammation resolution [15,16,17], and polyamine metabolism has been implicated in cellular stress adaptation and homeostatic regulation [21]. Accordingly, alterations in GUSB expression may influence multiple interconnected processes, including phagolysosomal turnover, metabolic adaptation, and inflammatory signaling.

To evaluate whether enhanced systemic exposure to CAZ-AVI can directly induce AKI, we adopted a dosing strategy based on the murine pharmacological data (Figure 1b). A dose of 625 mg/kg s.c., corresponding to high clinical exposure, was used as a reference. To model supratherapeutic exposure with levels exceeding those administered to patients with renal dysfunction, a 2-fold greater dose (1250 mg/kg) was used as the overdose condition. Mice received s.c. injections for 3 consecutive days once daily. Under these conditions, the 1250 mg/kg treated group exhibited a marked increase in serum BUN and creatinine compared with the 625 mg/kg group (Figure 1b, right panels), indicating that CAZ-AVI can induce AKI when systemic exposure exceeds clinically encountered levels.

To determine whether CAZ-AVI overexposure alters renal Gusb expression, we quantified whole-kidney Gusb mRNA levels across five murine AKI models (Figure 1c). At 48 h, CAZ-AVI treatment markedly reduced Gusb expression, and a similar decrease was observed following high-dose ATM-AVI administration. By contrast, cisplatin, rhabdomyolysis, and ischemia–reperfusion injury did not induce a reduction of similar magnitude under the experimental conditions used in this study. Together, these findings suggest that Gusb downregulation is associated with the β-lactam/β-lactamase inhibitor-related injury models examined here, rather than representing a universal response across all AKI models tested in this study.

Although the precise mechanism underlying Gusb downregulation remains unclear, prior studies have shown that renal β-glucuronidase (GUSB) expression is subject to genetic and transcriptional regulation, supporting the possibility that tubular injury or altered cellular states may influence Gusb expression. For example, Watson and Catterall demonstrated that mouse renal β-glucuronidase mRNA accumulation is genetically regulated (*Endocrinology*, 1986; PMID: 3004893), and Wawrzyniak et al. reported that multiple regulatory elements govern murine β-glucuronidase synthesis, including post-transcriptional mechanisms (*Genetics*, 1989; PMID: 2537250). These observations provide biological context for the variable Gusb expression patterns observed across AKI models in our study.

Immunofluorescence staining with a GUSB-specific antibody combined with lotus tetragonolobus lectin (LTL) co-labeling demonstrated that GUSB was abundantly expressed in the PTs at baseline (0 h). Following CAZ-AVI administration, GUSB transcription progressively declined, which was noticeable at 16 h; a marked loss of signal by 48 h was observed (Figure 1d). Consistent with this temporal decrease, periodic acid–Schiff (PAS) staining revealed a parallel increase in tubular injury scores and severe PT damage, evident at 48 h (Figure 1e). These findings indicate that GUSB downregulation is closely associated with the progression of CAZ-AVI-induced tubular injury.

Given that the loss of GUSB in PTs may accompany and potentially drive CAZ-AVI and ATM-AVI-induced nephrotoxicity, we generated PT-specific GUSB-deficient mice using a floxed Gusb allele crossed with a γ-glutamyl transpeptidase (γ-GT)-expressing Cre line. Immunofluorescence staining confirmed efficient PT-restricted knockdown of GUSB in the CKO kidneys, whereas robust GUSB expression was preserved in control littermates (Figure 1f). Histological analysis revealed significantly elevated tubular injury scores in CKO mice compared with controls (Figure 1g), accompanied by elevated serum BUN and creatinine levels (Figure 1h). These results demonstrate that Gusb deficiency is sufficient to induce proximal tubular injury and renal dysfunction, even in the absence of an exogenous injurious stimulus.

To elucidate the downstream pathways through which GUSB deficiency promotes AKI, we performed bulk RNA-seq analysis of whole-kidney tissues from control and CKO mice. Gene Ontology (GO) functional classification of differentially expressed genes revealed a prominent enrichment of efferocytosis-related pathways in CKO kidneys (Figure 1i). Consistently, Kyoto Encyclopedia of Genes and Genomes (KEGG) pathway analysis demonstrated a marked enrichment of phagocytosis-associated gene sets, with a high gene ratio and robust statistical significance (Figure 1j). Although GUSB is a classical lysosomal hydrolase, these transcriptomics data indicate that its loss primarily perturbs efferocytosis and phagocytosis programs rather than canonical lysosomal degradation pathways. Together, these findings suggest that the impaired clearance of apoptotic and damaged cell material represents a key molecular mechanism linking GUSB deficiency to tubular injury.

### 2.2. Gusb Deficiency in PTs Led to Marked Albuminuria and Ultrastructural Evidence of Efferocytosis

**Figure 2:**
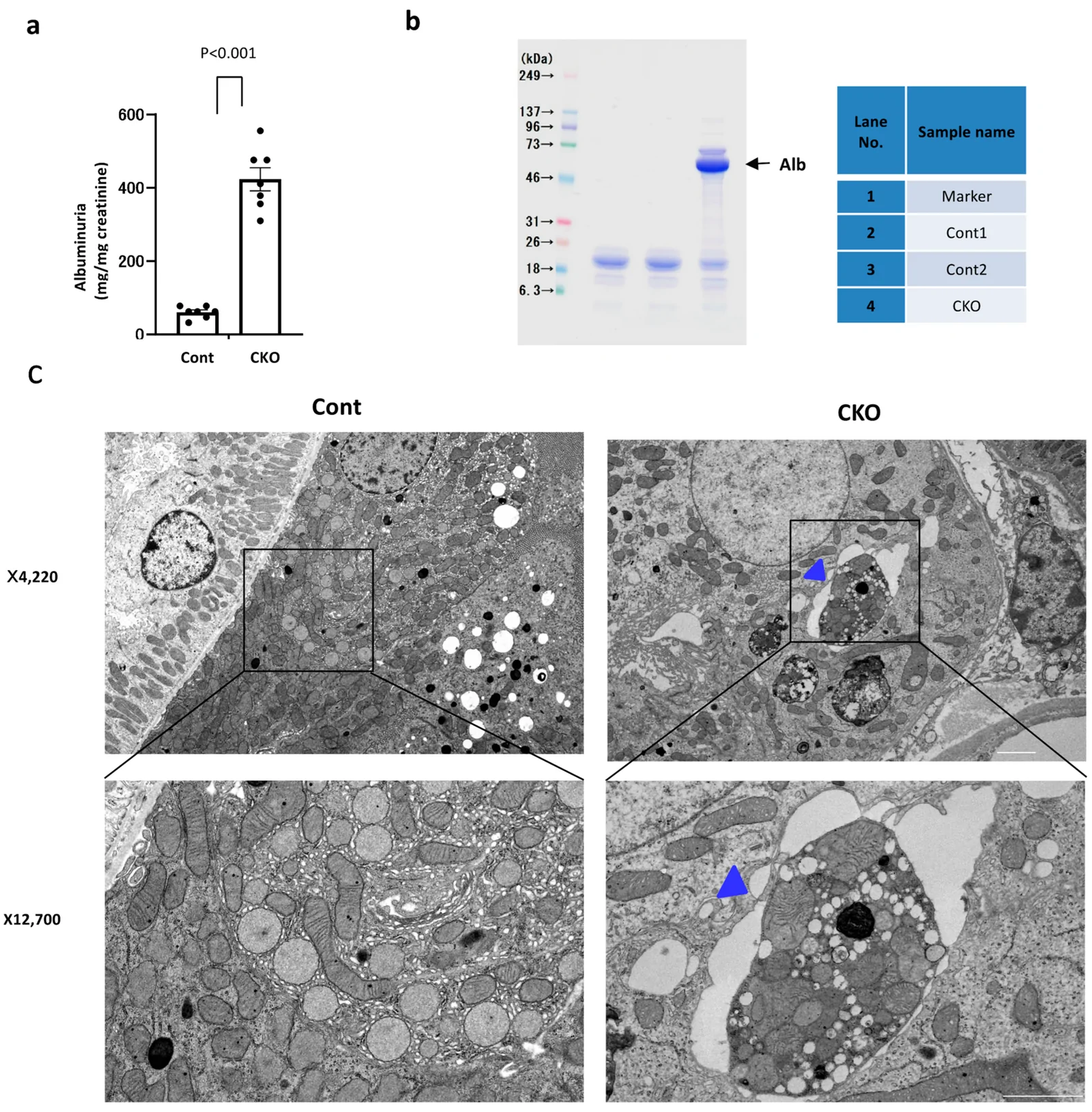
**Albuminuria and epithelial-to-epithelial efferocytosis in PT-specific Gusb CKO mice.** (**a**) Urinary albumin excretion in control (Cont) and Gusb conditional knockout (CKO) mice at 8 weeks of age (*n* = 7 per group). (**b**) Sodium dodecyl sulfate–polyacrylamide gel electrophoresis (SDS-PAGE) of urine samples from 8-week-old Cont and CKO mice. Urine was resolved on 15% SDS-PAGE and stained with Coomassie blue; it demonstrated a marked increase in albuminuria in CKO mice. (**c**) Representative transmission electron micrographs of proximal tubules (PTs) from Cont and CKO mice. In the kidneys of CKO animals, PT epithelial cells contained a single, large, phagocytic compartment (efferosome) engulfing degenerating cell material. At intermediate magnification (×4220), the engulfed cargo was observed to consist of collapsed nuclei, membrane fragments, and numerous mitochondria with well-preserved cristae, indicating that the phagocytosed material originated from neighboring PTs, uniquely enriched with mitochondria from the kidney. At high magnification (×12,700), electron-dense lysosomal granules and partially digested mitochondrial remnants were observed, consistent with phagosome–phagolysosome maturation. The absence of double-membrane autophagosomes and the presence of a single, mitochondria-rich cargo confirm that this structure represents true efferocytosis rather than autophagy or polyphagolysosome formation. Blue arrowheads indicate efferocytosis. Kidney tissues were embedded in Epon epoxy resin, and ten PTs per kidney were randomly imaged for morphometric evaluation. Scale bar: 50 nm. Data are presented as the means ± SEM and were analyzed using a two-tailed Student’s *t*-test. Exact *p*-values are indicated.

Compared with controls, CKO mice exhibited markedly elevated urinary albumin excretion (Figure 2a), confirmed by sodium dodecyl sulfate–polyacrylamide gel electrophoresis (SDS-PAGE) showing prominent albumin bands (Figure 2b). Since bulk RNA-seq analysis revealed a pronounced enrichment of efferocytosis-related genes in CKO kidneys, we next examined the morphological features of this process by transmission electron microscopy (TEM). At intermediate (×4220) and high (×12,700) magnifications, PT epithelial cells in CKO mice displayed large single phagocytic compartments containing degenerating cell fragments with collapsed nuclei, disrupted membranes, and numerous mitochondria with clearly preserved cristae (Figure 2c). At high magnification, the phagocytic compartments contained mitochondria-rich cellular fragments, a feature consistent with material originating from neighboring proximal tubules, which are uniquely enriched in mitochondria. The absence of double-membrane autophagosomes and the presence of a single, mature phagolysosomal compartment are morphological features compatible with efferocytic processing rather than canonical autophagy or polyphagocytosis. While these observations do not constitute a definitive tubule-to-tubule efferocytosis assay, they support the possibility that proximal tubules may internalize and degrade neighboring tubular fragments under conditions of CAZ-AVI-induced injury.

### 2.3. Gusb Deficiency Aggravates Tubular Injury Through Efferocytosis Failure, Polyphagolysosome Accumulation, Apoptosis, and Oxidative Stress

**Figure 3:**
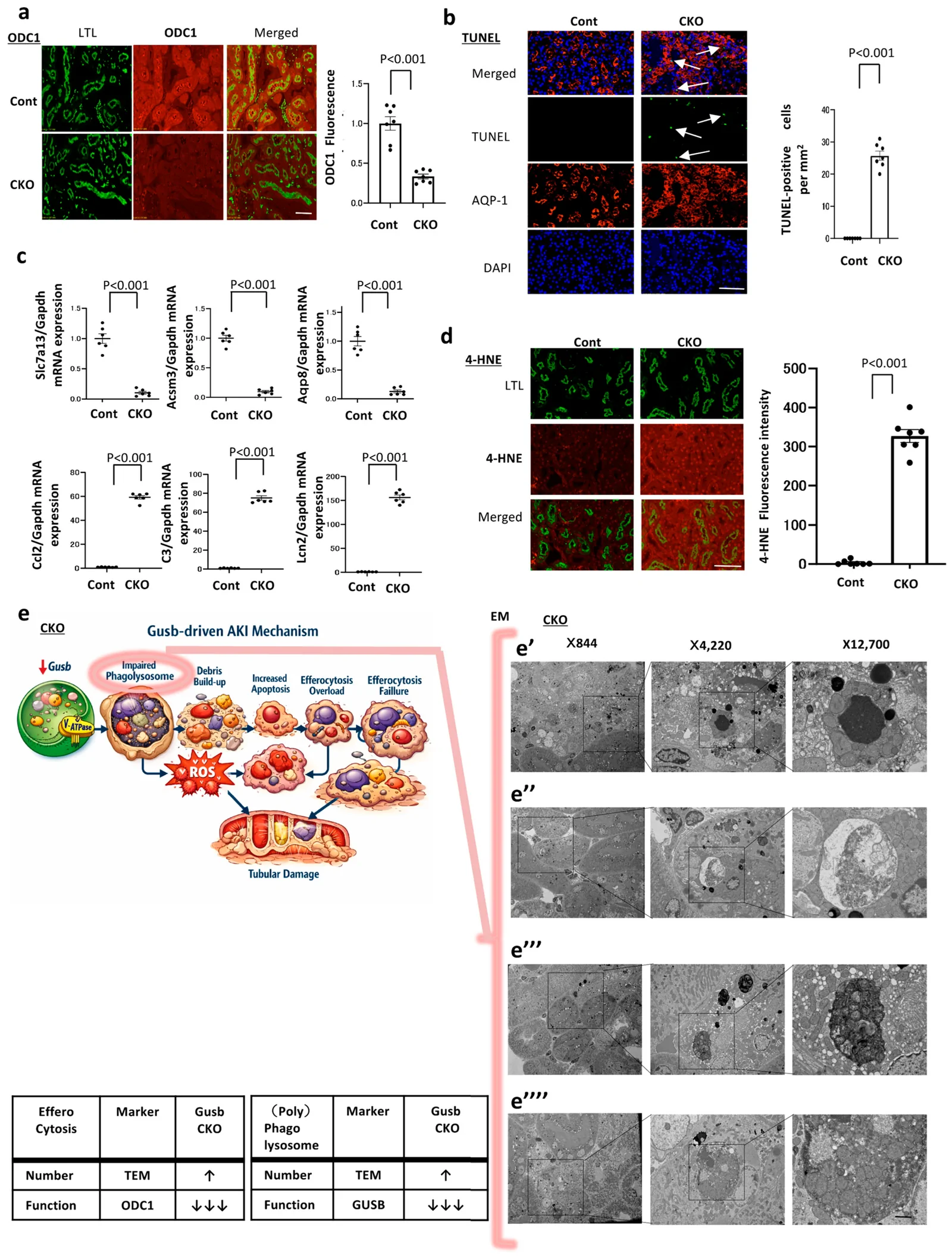
**Effects of PT-specific Gusb knockdown on efferocytosis function and the localized production of ROS.** (**a**) Representative immunofluorescence images showing the efferocytosis-related marker ODC1 in control (Cont) and Gusb conditional knockout (CKO) mice (*n* = 7 per group). LTL (green) marks proximal tubules (PTs); ODC1 (red) colocalized with LTL in Cont kidneys but was remarkably reduced in CKO kidneys. Quantitative fluorescence analysis is shown on the right (*p* < 0.001). (**b**) Representative double immunofluorescence of kidney sections from Cont and CKO mice (*n* = 7 per group) for TUNEL (red, apoptotic cells) and AQP-1 (green, PT marker). White arrowheads indicate apoptotic tubular cells. Quantification of TUNEL-positive cells per field is shown (*p* < 0.001). (**c**) Real-time quantitative PCR analysis of the mRNA levels of six efferocytosis-related renal genes in Cont and CKO mice (*n* = 6 per group). Slc7a13, Acsm3, and Aqp8 were significantly downregulated, whereas Ccl2, C3, and Lcn2 were upregulated in CKO kidneys (all *p* < 0.001). Data are presented as the mean ± SEM; statistical significance was determined via a two-tailed Student’s *t*-test. (**d**) Representative immunofluorescence for 4-hydroxynonenal (4-HNE), a marker of ROS, in Cont and CKO kidneys (*n* = 7 per group). LTL (green) identifies PTs; 4-HNE (red) is markedly enhanced in CKO kidneys. Quantitative fluorescence intensity is shown (*p* < 0.001). (**e**) Mechanistic schema (upper left) illustrating the proposed Gusb-driven AKI mechanism: Gusb deficiency impairs phagolysosomal acidification (via V-ATPase dysfunction), leading to debris accumulation, increased apoptosis, efferocytosis overload and failure, excessive ROS generation, and progressive tubular damage. TEM panels (**a**–**d**) show representative ultrastructural morphologies of giant polyphagolysosomes observed in CKO PTs. (**e′**) A single enlarged phagolysosome captured from low to high magnification containing dense bodies, membrane fragments, small vesicles, and mitochondrial remnants with preserved cristae. Absence of nuclear remnants, cytoplasmic continuity, or double-membrane phagosomes confirmed a mature phagolysosome overloaded with incompletely digested material. (**e′′**) Multiple enlarged phagolysosomes within intact proximal tubular cells displayed a mosaic mixture of mitochondrial fragments, multilamellar membranes, and dense bodies. Lack of cell-in-cell morphology excluded efferocytosis. (**e′′′**) Large membrane-bound compartments exhibited a characteristic swirl pattern formed by multilayered membrane fragments and partially digested organelles—a hallmark of phagolysosomal overload not seen in efferocytosis. (**e′′′′**) Ultra-high-resolution images showed dense accumulations of multilamellar bodies, membrane whorls, and mitochondrial cristae remnants, indicating the impaired degradation of PT-derived organelles. Together, these ultrastructural findings demonstrate that Gusb deficiency causes phagolysosomal dysfunction and the accumulation of giant polyphagolysosomes, providing a mechanistic basis for efferocytosis failure, apoptosis accumulation, and ROS elevation in Gusb-deficient PTs. Scale bars: 100 µm (immunofluorescence) and 50 nm (TEM). Abbreviations: AQP-1, aquaporin-1; LTL, lotus tetragonolobus lectin; ROS, reactive oxygen species; TUNEL, terminal deoxynucleotidyl transferase-mediated digoxigenin–deoxyuridine nick-end labeling. Exact *p*-values are indicated.

To elucidate how Gusb deficiency drives PT injury, we examined the changes in efferocytosis-related pathways. Bulk RNA-seq analysis (Table 3 and Table 4) revealed a downregulation of multiple efferocytosis-related functional markers in CKO kidneys, with a prominent decline in ornithine decarboxylase 1 (Odc1) (Table 4 and Supplementary Figure S1). Although Odc1 has been characterized as a macrophage-specific enzyme essential for sustaining continual efferocytosis [24], immunofluorescence staining demonstrated that in the kidney, it is prominently and constitutively expressed in PTs rather than macrophages. Under basal conditions, Odc1 colocalized with LTL, whereas its expression decreased markedly in CKO mice (Figure 3a). Thus, this study is the first to provide evidence that Odc1 plays a critical, epithelial-intrinsic role in PT efferocytosis, distinct from its established function in macrophages.

Despite the numerical increase in efferocytosis-related structures observed by TEM, the marked decline in Odc1 suggested that PT efferocytosis was functionally impaired. To evaluate the upstream efferocytosis triggers, we assessed apoptosis employing TUNEL staining. A large number of TUNEL-positive cells were detected within the AQP-1-positive PT region of CKO kidneys (Figure 3b), indicating a substantial increase in tubular apoptosis. These findings were consistent with the severe tubular injury detected by PAS staining (Figure 1g), suggesting that excessive apoptosis contributes to impaired albumin reabsorption and albuminuria development (Figure 2a,b).

Next, we substantiated the RNA-seq data by employing real-time PCR, focusing on genes related to PT function and efferocytosis (Figure 3c). The upregulated genes (Table 3) included Ccl2, C3, and Lcn2 (NGAL), indicating that injured PTs actively recruit inflammatory cells (Ccl2), opsonize debris (C3), and experience pronounced cell-level stress (Lcn2). Conversely, Slc7a13 (AGT1), Acsm3, and Aqp8 were significantly suppressed, reflecting the loss of antioxidant defense (Slc7a13) (Supplementary Figure S2), mitochondrial metabolic failure (Acsm3), and epithelial polarity (Aqp8). These results corroborate the transcriptomics data and demonstrate that Gusb-deficient PTs undergo inflammatory activation, metabolic collapse, and structural disintegration.

Because excessive apoptosis and impaired efferocytosis might enhance intracellular oxidative stress, we quantified the reactive oxygen species (ROS) marker 4-hydroxynonenal (4-HNE). Immunofluorescence staining revealed a marked increase in 4-HNE levels in CKO kidneys (Figure 3d). Collectively, these findings indicate that Gusb deficiency is accompanied by reduced Odc1 expression in proximal tubules and by morphological and functional features consistent with efferocytosis overload, including accumulation of apoptotic cells and elevated ROS levels. These observations suggest that altered Odc1-associated metabolic status may contribute to impaired phagolysosomal processing in Gusb-deficient PTs, ultimately coinciding with albuminuria and progressive tubular injury. However, the causal relationship between Odc1 downregulation and defective efferocytosis remains to be determined.

### 2.4. Gusb Deficiency Induces Phagolysosomal Dysfunction and Accumulation of Giant Polyphagolysosomes in PTs

Serial TEM analyses provided definitive evidence that Gusb deficiency causes profound phagolysosomal dysfunction in PTs (Supplementary Figure S3). Four representative categories of abnormal phagolysosomal structures were consistently observed (Figure 3(e′–e′′′′)).

(e′) Images from low to high magnification capture a single compartment, containing heterogeneous degradation products, such as dense bodies, membrane fragments, small vesicles, and mitochondrial remnants with preserved cristae. The absence of nuclear remnants, cytoplasmic continuity, or double-membrane phagosomes indicated a mature phagolysosome overloaded with incompletely digested material rather than an efferocytic event.

(e′′) Images show multiple enlarged phagolysosomes within an otherwise intact PT cell. Higher magnifications revealed a mosaic mixture of mitochondrial fragments, multilamellar membranes, and dense bodies. The absence of cell-in-cell morphology or any recognizable apoptotic cell architecture confirmed that these structures represented degraded organelles rather than engulfed apoptotic cells.

(e′′′) Images demonstrated several large, membrane-bound compartments exhibiting a characteristic swirl pattern formed by fragments of multilayered membranes and partially digested organelles. Such a pattern is a hallmark of phagolysosomal overload, arising when membrane debris accumulation outpaces degradation. Such structures were never observed during efferocytosis, where the ingested cell retains identifiable nuclear or cytoplasmic organization.

(e′′′′) Ultra-high-resolution images revealed a dense accumulation of multilamellar bodies, membrane whorls, and mitochondrial cristae remnants—organelles highly resistant to lysosomal degradation, thereby confirming the phenotype observed. The persistence of cristae strongly suggested that the engulfed material originated from PTs, which have the maximal mitochondrial density within the kidneys.

Taken together, these four categories of serial TEM images demonstrate a consistent pattern wherein Gusb deficiency disrupts phagolysosomal degradation capacity in PTs, inducing the accumulation of undigested cell debris and the formation of giant polyphagolysosomes. This phagolysosomal overload is parallel with efferocytosis failure (Figure 3a–d), increased apoptosis, and elevated ROS levels, establishing a mechanism underlying the progressive injury of PTs under Gusb-deficient conditions.

To determine whether CAZ-AVI-induced acute kidney injury exhibits dose dependency, we administered CAZ-AVI at 125, 625, or 1250 mg/kg/day for three consecutive days. At the clinically relevant dose of 125 mg/kg, serum BUN and creatinine remained within the normal range, comparable to untreated mice. In contrast, 625 mg/kg (approximately fivefold the therapeutic exposure) resulted in a moderate elevation of BUN and creatinine, whereas 1250 mg/kg induced the marked renal dysfunction observed in the main figure. These findings demonstrate a clear dose-dependent increase in biochemical AKI severity (Supplementary Figure S4a).

Histological evaluation using PAS staining further supported this dose dependency. No tubular injury was observed at 125 mg/kg, whereas 625 mg/kg produced moderate proximal tubular damage. At 1250 mg/kg, kidneys exhibited severe epithelial lysis and tubular denudation, consistent with the biochemical abnormalities and the injury pattern shown in the main figure. Thus, CAZ-AVI-induced AKI severity increases proportionally with drug dosage (Supplementary Figure S4b).

To validate the generation of proximal tubule-specific Gusb conditional knockout mice, we performed PCR-based genotyping for the Gusb floxed allele and the γ-GT–Cre transgene. PCR amplification of genomic DNA extracted from tail tissues successfully distinguished the wild-type Gusb allele (82 bp) from the floxed allele (133 bp), enabling clear identification of wild-type, heterozygous, and homozygous floxed mice (Supplementary Figure S5a).

PCR analysis of the γ-GT–Cre transgene generated the expected 411 bp fragment, confirming Cre-positive (Cre/+) and Cre-negative (+/+) genotypes within representative litters (Supplementary Figure S5b).

Crossing Gusb flox/flox mice with γ-GT–Cre mice yielded offspring in which the floxed allele appeared as a double band in heterozygous mice (fl/+) and a single 133 bp band in homozygous floxed mice (fl/fl). Mice carrying the fl/fl; Cre/+ genotype were designated as proximal tubule-specific Gusb CKO mice, whereas fl/fl littermates served as controls. These results confirm the successful generation and validation of the Gusb CKO model (Supplementary Figure S5c).

To determine whether CAZ-AVI–induced acute kidney injury is accompanied by glomerular dysfunction, we first quantified plasma albumin concentrations in control and Gusb CKO mice. Plasma albumin levels remained unchanged between groups, indicating that CAZ-AVI at 1250 mg/kg/day does not induce systemic hypoalbuminemia. These findings suggest that the albuminuria observed in this AKI model is attributable to impaired proximal tubule albumin endocytosis rather than podocyte or glomerular barrier injury (Supplementary Figure S6a).

To further evaluate glomerular structural integrity, we examined the expression of podocyte markers Nephrin and Podocin. Immunofluorescence analysis demonstrated that Nephrin-positive and Podocin-positive areas were preserved in CAZ-AVI–treated mice compared with controls, with no reduction in fluorescence intensity or glomerular localization. These results support the conclusion that podocyte injury does not occur in CAZ-AVI–induced AKI, consistent with the maintained plasma albumin levels (Supplementary Figure S6b,c).

To validate that apoptotic cells undergoing efferocytosis originate from proximal tubules, we performed dual-label immunofluorescence staining using three independent proximal tubule markers—AQP1, LTL, and Megalin—together with TUNEL. In CAZ-AVI–treated mice (1250 mg/kg/day), TUNEL-positive apoptotic cells consistently colocalized with AQP1-positive proximal tubules, confirming that apoptotic cells arise within the proximal tubular compartment (Supplementary Figure S7a).

Similarly, TUNEL-positive cells were detected within LTL-positive tubules, demonstrating that apoptotic cells reside in brush-border-bearing proximal tubules (Supplementary Figure S7b).

Finally, Megalin-positive proximal tubules also contained TUNEL-positive apoptotic cells, further supporting that the apoptotic population originates from proximal tubule epithelial cells (Supplementary Figure S7c).

Together, these findings validate that the cells undergoing apoptosis—and subsequently becoming targets for efferocytosis—are proximal tubule cells, as demonstrated by three independent PT markers.

To further validate that apoptotic cells in CAZ-AVI–induced acute kidney injury originate from proximal tubules, we used SLC34a1-GCE and Rosa26-tdTomato lineage-tracing mice, in which proximal tubule epithelial cells are permanently labeled following tamoxifen-induced Cre activation. In CAZ-AVI-treated mice (1250 mg/kg/day), TUNEL-positive apoptotic cells consistently colocalized with tdTomato-labeled proximal tubule cells, demonstrating that the apoptotic population arises directly from the proximal tubular lineage. Quantitative analysis confirmed that a subset of tdTomato-positive proximal tubule cells underwent apoptosis, supporting the conclusion that the cells targeted for efferocytosis in this injury model are derived from the proximal tubule compartment (Supplementary Figure S8).

### 2.5. PT-Specific Gusb Overexpression Protects Against CAZ-AVI-Induced AKI

**Figure 4:**
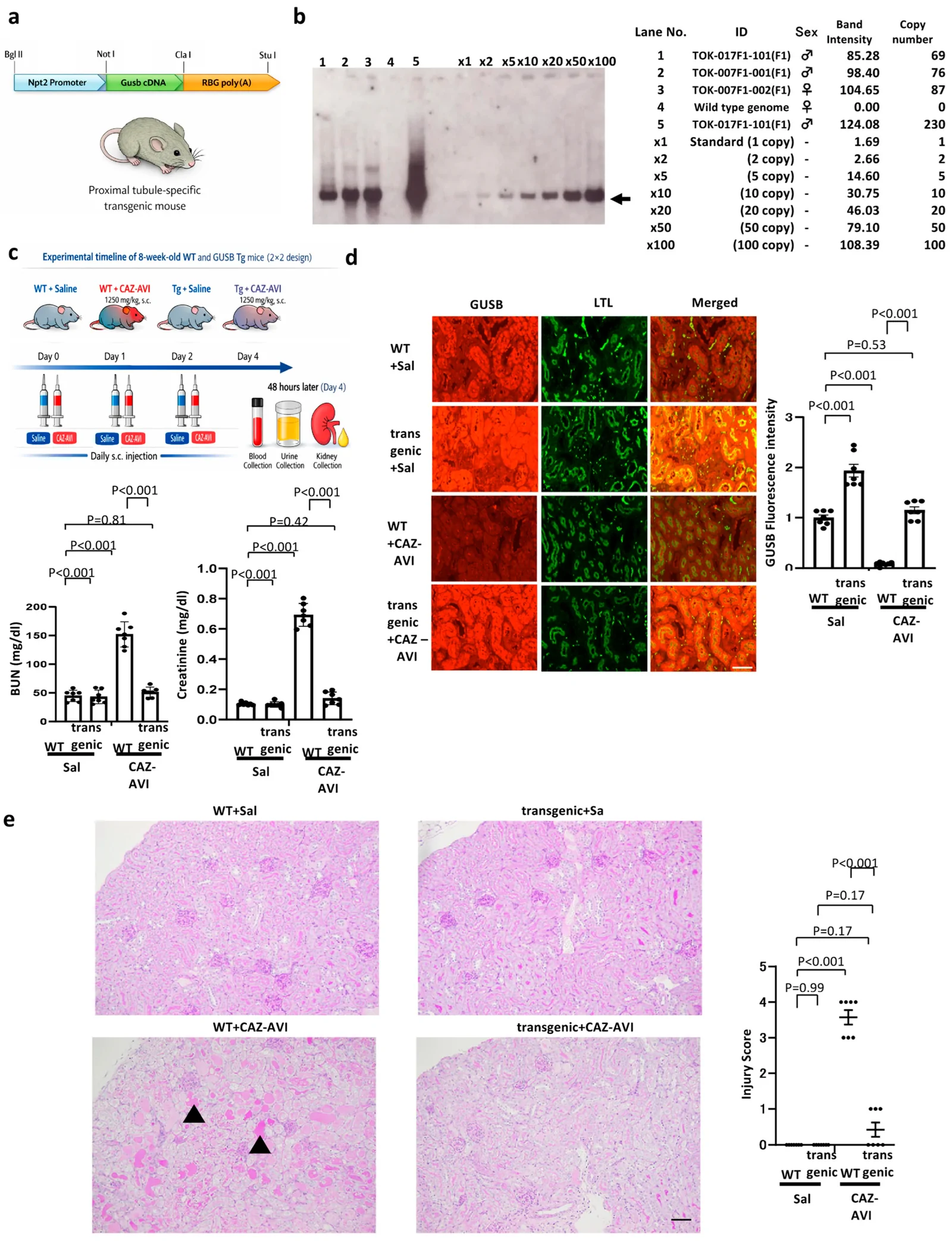
**Breeding and anti-AKI phenotypes of transgenic mice.** (**a**) Constructs used to generate transgenic mice. The transgene fragment consisted of the Npt2 promoter, murine Gusb cDNA, and RBG poly(A) sequences for PT-specific expression. (**b**) Southern blots indicating the copy number of the Gusb transgene in each mouse line (arrows indicate bands corresponding to transgene-derived Gusb). Band intensity of 85.28 in the TOK-017F1-101 line was consistent with a high-copy transgene (approximately 70–80 copies). (**c**) Experimental design of the CAZ-AVI-induced AKI model. Eight-week-old wild-type (WT) and Gusb transgenic mice were subcutaneously administered CAZ-AVI at 1250 mg/kg/day for three consecutive days, or saline as a control. At 48 h after the final injection, blood, urine, and kidney tissues were collected for analysis. The bottom graphs show serum BUN and creatinine levels in the four experimental groups: WT + saline, WT + CAZ-AVI, transgenic + saline, and transgenic + CAZ-AVI. (**d**) Representative immunofluorescence images of kidney cryosections from each group of animals at 8 weeks of age. GUSB (red) and LTL (green) staining demonstrated reduced GUSB expression in WT + CAZ-AVI kidneys, whereas transgenic mice maintained high GUSB expression under saline and CAZ-AVI conditions. (**e**) Representative PAS-stained kidney sections and corresponding tubular injury scores for the four groups. WT + CAZ-AVI kidneys exhibited marked tubular epithelial swelling, loss of brush border, and vacuolization, whereas transgenic + CAZ-AVI kidneys showed a significant attenuation of injury. Scale bars: 100 µm (immunofluorescence) and 50 µm (light microscopy). Data were analyzed using one-way ANOVA with Bonferroni correction. Abbreviations: RBG, rabbit β-globulin; LTL, lotus tetragonolobus lectin; BUN, blood urea nitrogen; Cr, creatinine. Exact *p*-values are indicated.

To examine whether enhanced Gusb expression mitigates CAZ-AVI-induced tubular injury, we generated PT-specific Gusb transgenic mice. As shown in Figure 4a, the transgene construct consisted of Gusb cDNA driven by the Npt2 promoter attached to an RBG poly(A) tail, enabling PT-specific expression. Southern blot analysis (Figure 4b) confirmed transgene integration, with established lines carrying approximately 50–100 copies.

Eight-week-old wild-type (WT) and Gusb transgenic mice were divided into four treatment groups that received s.c. injections daily for 3 days: WT + saline, WT + CAZ-AVI, transgenic + saline, and transgenic + CAZ-AVI (Figure 4c). WT mice developed AKI following CAZ-AVI exposure, as evidenced by marked increases in serum BUN and creatinine contents. In contrast, transgenic mice maintained normal renal function, indicating that Gusb overexpression effectively prevented CAZ-AVI-induced functional decline.

Immunofluorescence revealed that GUSB expression was substantially reduced in WT kidneys after CAZ-AVI administration, whereas transgenic mice retained higher GUSB fluorescence intensity under saline and CAZ-AVI conditions (Figure 4d). These results indicate that enforced GUSB expression stabilizes proximal tubular GUSB levels even under pharmacologic stress.

Histological evaluation showed severe tubular epithelial swelling, brush-border loss, and vacuolization in WT kidneys exposed to CAZ-AVI, while transgenic kidneys exhibited preserved tubular architecture and minimal injury (Figure 4e). Quantitative scoring confirmed substantial tissue protection in the transgenic group.

Collectively, these findings demonstrate that PT-specific Gusb overexpression robustly protects against CAZ-AVI-induced AKI, preserving renal function and tubular structure. Mechanistically, sustained Gusb expression appears to normalize phagolysosomal function and suppress ROS generation and apoptosis. This phenomenon is the inverse of efferocytosis failure and phagolysosomal dysfunction (Figure 3a–d), underscoring the central role of Gusb in maintaining proximal tubular resilience under drug-induced stress.

### 2.6. Protective Effects of Gusb-Transgenic Mice on Albuminuria and Efferocytosis

**Figure 5:**
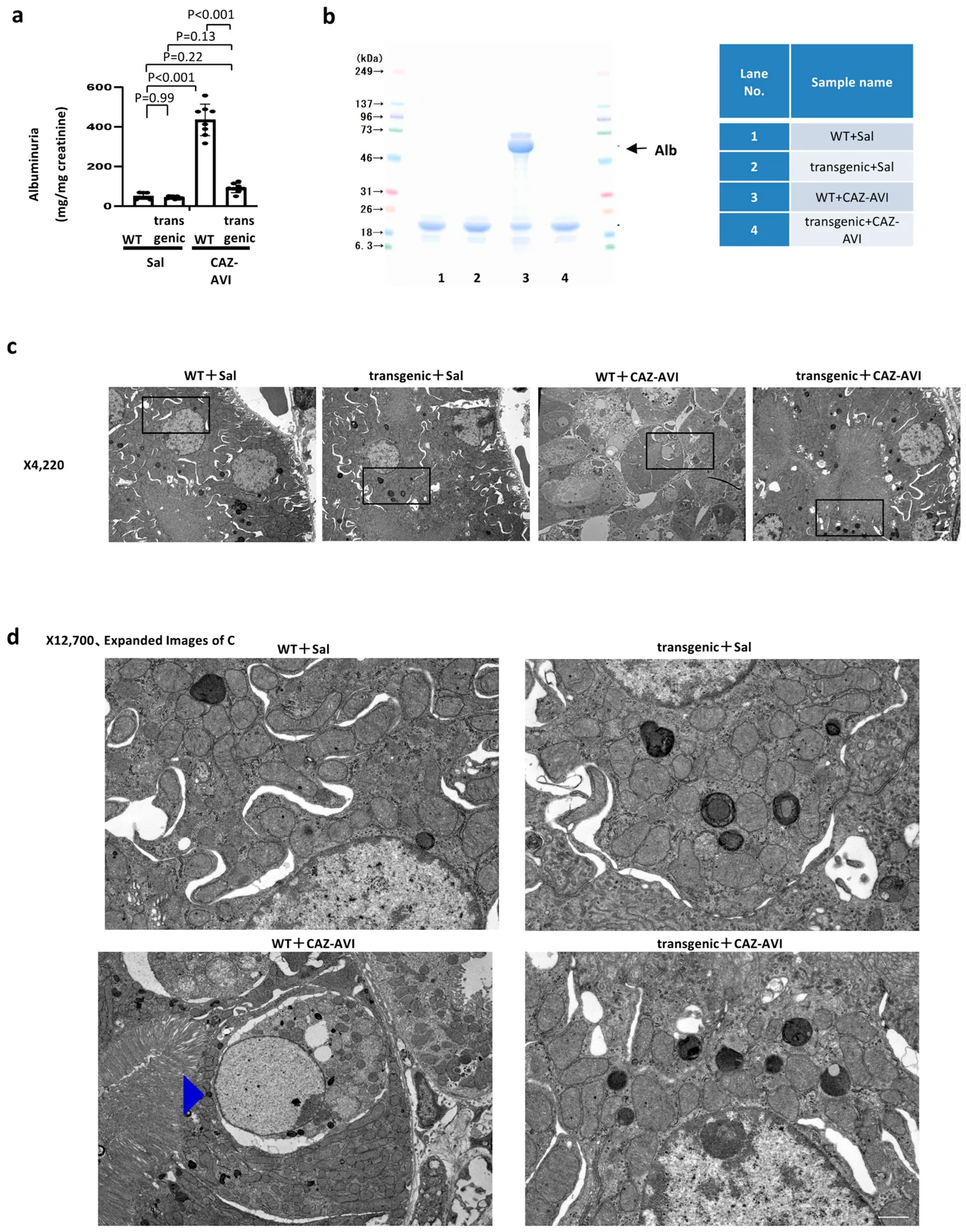
**Anti-efferocytosis phenotypes of Gusb transgenic mice.** (**a**) Urinary albumin excretion in the four groups of 8-week-old mice. (**b**) SDS-PAGE analysis of urine sampled from the four groups of mice. Urinary proteins were separated by 15% SDS-PAGE and stained with Coomassie blue. (**c**) Representative transmission electron micrographs (×4220) of PTs from each group (black squares indicate enlarged regions). In WT + CAZ-AVI kidneys, PT epithelial cells exhibited clear efferocytosis, characterized by a viable host PT cell containing a large, heterogeneous structure composed of fragmented cell debris. (**d**) High-magnification images (×12,700) from panel (**c**). Blue arrowheads point to efferocytic phagosomes. The engulfed material lacked nuclear continuity and chromatin but instead contained mitochondrial fragments with preserved cristae, membrane remnants, and electron-dense granules—morphological features that definitively identify the engulfed cell as a neighboring PT epithelial cell, establishing that PTs can engulf and digest other PTs. Such efferocytic structures were absent in Gusb transgenic + CAZ-AVI mice. Kidney tissues were embedded in Epon epoxy resin for electron microscopy (EM). For each mouse, ten PTs were randomly imaged to evaluate tubular morphometry. Each group consisted of seven mice. Scale bar: 500 nm (EM). Data were analyzed using one-way ANOVA with Bonferroni correction. Exact *p*-values are shown.

CAZ-AVI-induced albuminuria increased markedly in WT + CAZ-AVI mice but was significantly suppressed in Gusb transgenic + CAZ-AVI mice (Figure 5a). SDS-PAGE confirmed an intense 66 kDa albumin band in the urine samples of WT + CAZ-AVI animals, whereas transgenic + CAZ-AVI mice demonstrated minimal albumin excretion (Figure 5b). At the ultrastructural level, WT + CAZ-AVI kidneys presented definitive evidence of efferocytosis within PTs (Figure 5c). TEM at ×4220 revealed a proximal tubular epithelial cell acting as the host, maintaining an intact nucleus, cytoplasm, and mitochondria. Within the same cell, a large, heterogeneous structure representing engulfed debris from a dying neighboring PT cell was detected. The material lacked nuclear continuity and chromatin but consisted of mitochondrial fragments with preserved cristae, membrane remnants, and electron-dense granules—a pattern incompatible with autophagy and fully consistent with efferocytosis uptake of an apoptotic PT cell. Higher-magnification TEM (×12,700) demonstrated phagosomelysosome fusion, with electron-dense lysosomal granules and partially digested membranes, indicating progression to the phagolysosomal stage of efferocytosis. The presence of mitochondrial fragments with intact cristae within the cargo provides direct morphological evidence that the engulfed cell was a PT epithelial cell, establishing the previously unrecognized phenomenon that “PTs can engulf and digest neighboring PTs.” In contrast, no efferocytic structures were observed in Gusb transgenic + CAZ-AVI mice, whose PTs retained a normal ultrastructure. In summary, these findings demonstrate that Gusb overexpression protects against CAZ-AVI-induced albuminuria and pathological PT-to-PT efferocytosis, thereby preserving proximal tubular integrity.

### 2.7. Gusb Overexpression Prevents PT Self-Destruction and Phagolysosomal Failure Under CAZ-AVI Exposure

To determine how Gusb overexpression modulates PT responses to CAZ-AVI, we examined efferocytosis-related metabolism, apoptosis, PT-intrinsic gene-expression programs, oxidative stress, and ultrastructural changes.

**Figure 6:**
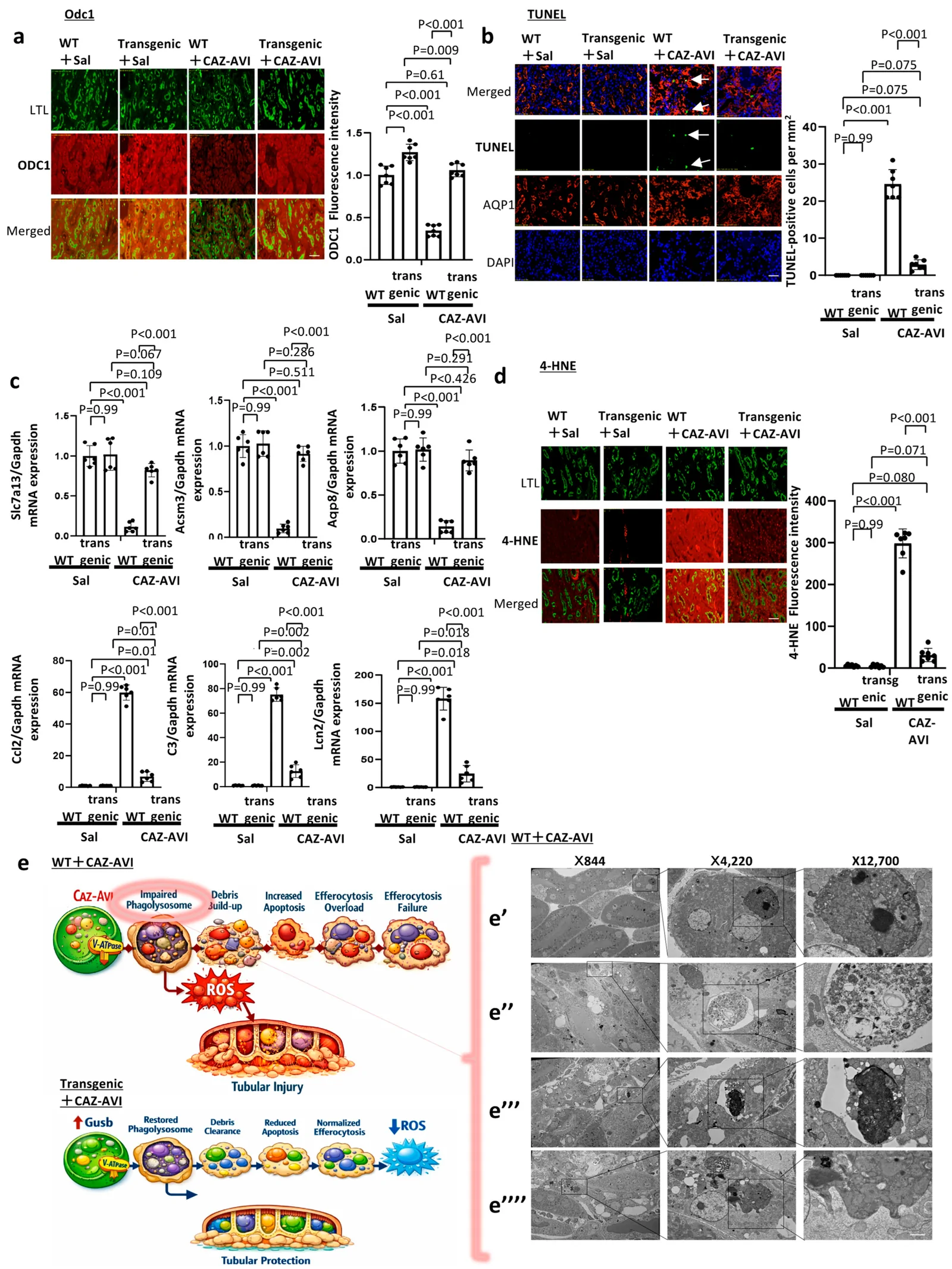
**Protective effects of Gusb overexpression on phagolysosomal function and PT injury under CAZ-AVI exposure.** (**a**) Representative immunofluorescence images showing Odc1 (red) colocalized with the PT marker LTL (green) and PMP70. Odc1 was markedly reduced in WT + CAZ-AVI kidneys, indicating impaired efferocytosis-related metabolic capacity of PTs. Quantification is shown on the right (*n* = 7 mice/group). (**b**) TUNEL staining (red) revealed abundant apoptotic tubular cells in WT + CAZ-AVI kidneys, whereas transgenic + CAZ-AVI mice showed markedly fewer apoptotic cells. Arrows indicate TUNEL-positive cells. Quantification is presented on the right (*n* = 7 mice/group). (**c**) Real-time PCR analysis of six PT-intrinsic genes associated with efferocytosis overload and AKI (Ccl2, C3, Lcn2, Slc7a13, Acsm3, and Aqp8). In WT + CAZ-AVI kidneys, Ccl2, C3, and Lcn2 were strongly induced, reflecting monocyte/macrophage recruitment (Ccl2), complement-mediated opsonization of PT-derived debris (C3), and severe tubular stress (Lcn2). Conversely, Slc7a13, Acsm3, and Aqp8 were downregulated, indicating a loss of antioxidant defense (Slc7a13), mitochondrial metabolic failure (Acsm3), and lack of epithelial polarity/identity (Aqp8). All six genes were normalized in transgenic + CAZ-AVI kidneys (*n* = 6 mice/group). (**d**) Immunostaining for 4-HNE (red) demonstrated robust ROS accumulation in WT + CAZ-AVI kidneys, predominantly within the PTs. In contrast, staining was weak in WT + saline animals and was markedly suppressed in transgenic + CAZ-AVI kidneys (*n* = 7 mice/group). Fluorescence intensity was quantified and is presented in the lower panel. Data were analyzed using one-way ANOVA with Bonferroni correction. (**e**) Left: Schematic illustration summarizing the proposed mechanism. WT + CAZ-AVI kidneys exhibited a pathological pattern resembling that of Gusb CKO mice, including phagolysosomal dysfunction, apoptosis, and efferocytosis overload, whereas such abnormalities were negligible in transgenic + CAZ-AVI mice. Right: Representative TEM images (**a**–**d**) showing polyphagolysosomes in WT + CAZ-AVI kidneys. (**e′**) A single enlarged phagolysosome containing dense bodies, membrane fragments, small vesicles, and mitochondrial remnants with preserved cristae, without nuclear remnants, cytoplasmic continuity, or double-membrane phagosomes. (**e′′**) Multiple enlarged phagolysosomes within an intact proximal tubular cell, containing mitochondrial fragments, multilamellar membranes, and dense bodies, with no evidence of cell-in-cell structures. (**e′′′**) Large membrane-bound compartments exhibited a swirl pattern formed by multilayered membrane fragments and partially digested organelles—a hallmark of phagolysosomal overload. (**e′′′′**) Ultra-high-resolution images showed multilamellar bodies, membrane whorls, and mitochondrial cristae remnants, indicating impaired degradation of PT-derived organelles. Such abnormal structures were absent in transgenic + CAZ-AVI kidneys, indicating preserved phagolysosomal integrity. Scale bars: 100 µm (immunofluorescence) and 50 nm (TEM). Exact *p*-values are shown.

Immunofluorescence for Odc1 showed that Odc1 expression was markedly reduced in the kidneys of WT + CAZ-AVI animals, whereas those from transgenic + CAZ-AVI mice maintained strong Odc1 signals (Figure 6a). Odc1 colocalized with LTL, indicating that efferocytosis failure was specific to PTs and that Gusb overexpression preserved efferocytic metabolic capacity.

TUNEL staining revealed an abundance of apoptotic cells in WT + CAZ-AVI kidneys, while markedly fewer apoptotic cells were seen in transgenic + CAZ-AVI kidneys (Figure 6b). Colocalization with aquaporin-1 confirmed that apoptosis was almost exclusive to PTs, consistent with the concept that both the engulfing and engulfed cells are PTs (Figure 5).

Gene expression analysis demonstrated that Ccl2, C3, and Lcn2 were strongly upregulated while Slc7a13, Acsm3, and Aqp8 were suppressed in WT + CAZ-AVI kidneys (Figure 6c). These changes indicate that PTs simultaneously recruit monocytes/macrophages (Ccl2), opsonize the surrounding debris (C3), signal injury (Lcn2), and lose antioxidant, metabolic, and epithelial identity programs (Slc7a13, Acsm3, and Aqp8). This pattern reflects that PTs self-destruct under efferocytosis overload, whereas transgenic + CAZ-AVI kidneys showed near-complete normalization of all six genes, indicating the preservation of PT homeostasis.

Oxidative stress analysis showed robust 4-HNE accumulation within WT + CAZ-AVI kidneys, whereas transgenic + CAZ-AVI kidneys exhibited minimal 4-HNE signals (Figure 6d), indicating that Gusb overexpression prevents ROS amplification by maintaining phagolysosomal function.

TEM revealed numerous polyphagolysosomes in WT + CAZ-AVI kidneys but none in the transgenic + CAZ-AVI ones (Figure 6(e′–e′′′′)).

(e′) A single enlarged phagolysosome contained dense bodies, membrane fragments, small vesicles, and mitochondrial remnants with preserved cristae, without nuclear remnants, cytoplasmic continuity, or double-membrane phagosomes, indicating phagolysosomal overload rather than efferocytosis.

(e′′) Multiple enlarged phagolysosomes were present within intact proximal tubular cells, containing mitochondrial fragments, multilamellar membranes, and dense bodies, with no evidence of cell-in-cell structures.

(e′′′) Large membrane-bound compartments exhibited a swirl pattern formed by multilayered membrane fragments and partially digested organelles—a hallmark of phagolysosomal overload not observed during efferocytosis.

(e′′′′) Ultra-high-resolution images showed multilamellar bodies, membrane whorls, and mitochondrial cristae remnants, indicating an impaired degradation of PT-derived organelles.

These ultrastructural findings confirm that degraded material originated from PTs, extending the “PT eats PT” phenomenon (Figure 5) into a later stage of phagolysosomal overload. Their complete absence in transgenic + CAZ-AVI kidneys demonstrates preserved phagolysosomal integrity.

WT + CAZ-AVI kidneys exhibited a coherent pattern of PT-specific phagolysosomal failure across metabolic (Odc1), apoptotic (TUNEL), inflammatory/stress (Ccl2, C3, and Lcn2), protective/identity (Slc7a13, Acsm3, and Aqp8), oxidative (4-HNE), and ultrastructural (polyphagolysosome) analyses, closely resembling the Gusb-deficient phenotype (Figure 3a–d). Strikingly, Gusb overexpression prevented each abnormality, demonstrating comprehensive protection of PT phagolysosomal homeostasis.

## 3. Discussion

In this study, we identified Gusb as an essential regulator of PT homeostasis during antibiotic-induced cellular stress. Using inducible PT-specific Gusb-CKO and Gusb-Tg mice, we demonstrated that Gusb preserves phagolysosomal competence, prevents mitochondrial injury, and protects against CAZ-AVI-induced AKI. These findings provide a mechanistic framework that explains the emerging clinical observations of nephrotoxicity associated with CAZ-AVI, particularly in vulnerable populations like transplant recipients [25,26].

Exposure to CAZ-AVI in WT mice induced a characteristic accumulation of enlarged, electron-dense polyphagolysosomes within PT cells, accompanied by mitochondrial swelling, cristae rarefaction, and cytosolic ROS accumulation (Figure 5c). These ultrastructural abnormalities resemble the features of “degradation-phase failure” described in defective efferocytosis, wherein phagosomes fail to mature or fuse with lysosomes, causing secondary necrosis and inflammation amplification [27,28,29]. Efferocytosis-related degradation-phase failure has been described across multiple organ systems, including the cardiovascular system [30], liver [31], the central nervous system [32], and tumors [33], in which the impaired clearance of dying cells drives chronic inflammation [34,35,36] and maladaptive remodeling [37,38,39,40,41]. However, whether a similar mechanism operates in the kidney is yet to be explored.

Our data extend this paradigm to the kidney. Although PT cells are not typical phagocytes, they engage in efferocytic clearance of apoptotic neighbors during toxic stress, supported by the recognition of efferocytosis-related pathways during KEGG analysis (Figure 1b). Importantly, Gusb overexpression restored phagolysosomal morphology, reduced ROS accumulation, and preserved mitochondrial structure (Figure 4d,e), indicating that Gusb is a rate-limiting factor for the degradative capacity of lysosomes during high-burden efferocytosis.

Although CAZ-AVI is safer than polymyxins, real-world pharmacovigilance data indicate a measurable incidence of AKI. A recent FAERS analysis—a real-world study of adverse events associated with CAZ-AVI—reported significant renal safety signals, including AKI, renal failure, and elevated creatinine [8,9]. In liver transplant recipients, a study on the effectiveness and safety of CAZ-AVI vs. polymyxin B in LT recipients observed AKI in 50% of CAZ-AVI-treated patients, substantially higher than in non-transplant cohorts [7].

Our findings provide a mechanistic explanation for these clinical observations. CAZ-AVI induces tubular apoptosis (Figure 6a,b), increasing the efferocytic burden. When phagolysosomal capacity is overwhelmed, phagolysosome failure ensues, causing mitochondrial injury, ROS accumulation, and, ultimately, AKI. Gusb overexpression prevented such a cascade, normalizing efferocytosis and reducing tubular injury (Figure 6e). These results suggest that Gusb-dependent lysosomal resilience is a key determinant of susceptibility to CAZ-AVI nephrotoxicity.

Intriguingly, both CAZ-AVI and ATM-AVI reduced Gusb expression in vivo (Figure 1c), raising the possibility that AVI modulates lysosomal or efferocytic pathways. However, high-dose AVI alone did not induce AKI in our preliminary experiments, negating the nephrotoxic effects of the drug. Instead, a combination of AVI with β-lactams may alter phagolysosomal dynamics under stress.

Hitherto, no study has systematically quantified AKI incidence with ATM-AVI, although emerging reports suggest that combinations of β-lactam/β-lactamase inhibitors may share downstream effects on phagolysosomal pathways. Whether other combinations, such as ceftolozane–tazobactam or meropenem–vaborbactam, induce similar polyphagolysosomal stress remains unknown [7,8,9]. The current findings highlight the need for comparative mechanistic studies across β-lactamase inhibitor classes.

Transplant recipients represent a population in which CAZ-AVI is increasingly used; however, they are uniquely vulnerable to AKI due to the nephrotoxicity of calcineurin inhibitors, ischemia–reperfusion injury, heightened apoptotic turnover, and impaired mitochondrial reserve. In a liver transplant cohort, AKI occurred in 50% of CAZ-AVI-treated patients, which exceeded the proportion observed in general ICU populations [7]. Our data suggest that CAZ-AVI may act as a “second hit” in these patients by overwhelming tubular efferocytic capacity. Therefore, enhancing Gusb activity may represent a precision-medicine strategy to protect allografts during high-risk antimicrobial therapy.

Given the impracticality of renal biopsy during acute infection, non-invasive biomarkers are an urgent requirement. Based on our mechanistic findings and efferocytosis biology, promising candidates identified include urinary mitochondrial DNA [42], lysosomal hydrolases [43] (β-glucuronidase and cathepsins), LC3-II or Rubicon fragments [44], extracellular vesicles [45] containing undigested apoptotic cargo, and ROS-modified lipids such as 4-HNE (Figure 3d). Longitudinal profiling of CAZ-AVI-treated transplant recipients may reveal biomarker-specific signatures predictive of efferocytic overload and impending AKI.

The supplementary analyses presented in Supplementary Figures S4–S8 provide additional mechanistic support for the conclusion that CAZ-AVI-induced acute kidney injury (AKI) primarily affects proximal tubule (PT) epithelial cells and that the apoptotic cells undergoing efferocytosis originate from the PT compartment.

First, the dose–response experiments demonstrated that CAZ-AVI-induced AKI severity increases proportionally with drug exposure. At the clinically relevant dose of 125 mg/kg/day, biochemical and histological abnormalities were absent, whereas 625 mg/kg/day produced moderate renal dysfunction and 1250 mg/kg/day reproduced the severe injury pattern shown in the main figure (Supplementary Figure S4). These findings support the validity of the 1250 mg/kg/day model used throughout the study and indicate that CAZ-AVI nephrotoxicity follows a graded injury pattern.

Second, genotyping analyses verified the successful generation of the PT-specific Gusb conditional knockout (CKO) model. PCR assays reliably distinguished wild-type, floxed, and Cre-positive alleles, confirming the accuracy of the breeding strategy and ensuring that subsequent phenotypic analyses were performed in genetically validated CKO mice (Supplementary Figure S5). This genetic precision is essential for attributing the observed efferocytosis defects specifically to Gusb loss within PT cells.

Third, supplementary evaluation of glomerular integrity demonstrated that CAZ-AVI-induced AKI does not involve podocyte injury. Plasma albumin levels remained stable, and immunofluorescence staining for Nephrin and Podocin showed preserved glomerular architecture in CAZ-AVI-treated mice. These results indicate that the albuminuria observed in this model arises from impaired PT albumin endocytosis rather than glomerular barrier disruption (Supplementary Figure S6). This distinction is critical, as it confirms that the injury phenotype is localized to the PT and not confounded by upstream glomerular pathology.

Fourth, triple immunofluorescence staining using three independent PT markers—AQP1, LTL, and Megalin—demonstrated consistent colocalization of TUNEL-positive apoptotic cells with PT epithelia. These findings validate that apoptotic cells undergoing efferocytosis originate from PT cells and not from other nephron segments (Supplementary Figure S7). The use of multiple PT markers provides robust anatomical confirmation of the cellular origin of apoptosis.

Finally, lineage-tracing experiments using SLC34a1-GCE and Rosa26-tdTomato mice further strengthened this conclusion. In CAZ-AVI–treated mice, TUNEL-positive apoptotic cells consistently colocalized with tdTomato-labeled PT cells, demonstrating that the apoptotic population arises directly from the PT lineage. Quantitative analysis confirmed that a subset of lineage-labeled PT cells undergo apoptosis, providing definitive genetic evidence that the cells targeted for efferocytosis in this injury model are PT-derived (Supplementary Figure S8).

Collectively, these supplementary findings support the central conclusion of the study: CAZ-AVI-induced AKI is a dose-dependent, PT-specific injury in which apoptotic PT epithelial cells become targets for efferocytosis. The integration of biochemical, histological, immunofluorescent, and lineage-tracing evidence provides a comprehensive and internally consistent mechanistic framework that reinforces the main results.

A limitation of our proximal tubule-specific Gusb knockout model is that the γ-glutamyl transpeptidase (γ-GT)–Cre driver exhibits known activity in hepatocytes in addition to proximal tubules. Because the liver contributes to the disposition and systemic bioavailability of β-lactam/β-lactamase inhibitor combinations, hepatic Cre-mediated recombination may theoretically influence CAZ-AVI exposure and thereby modulate the severity of drug-induced AKI. Although no overt hepatic dysfunction was observed in our mice, this caveat should be considered when interpreting the results, and future studies using alternative PT-specific Cre drivers may help further refine the model.

Our study identifies Gusb as a critical regulator of tubular efferocytosis and phagolysosomal homeostasis during CAZ-AVI-induced stress. The inability to model this process in vitro underscores the necessity of in vivo systems to study epithelial phagocytic biology. The current findings provide a mechanistic explanation for emerging clinical reports of CAZ-AVI-associated AKI and suggest that enhancing Gusb activity may represent a novel therapeutic strategy to protect vulnerable kidneys—particularly in transplant recipients. Thus, future studies should focus on developing Gusb-targeted interventions and validating non-invasive biomarkers of efferocytic failure in human cohorts.

## 4. Materials and Methods

The methodology is detailed in the Supplementary Materials.

### 4.1. Transgenic and CKO Mice

The breeding of PT-specific CKO mice and three types of control mice is outlined in Table 1.

### 4.2. Mouse Models of AKI

Under laboratory conditions, healthy, well-nourished 8-week-old male C57BL/6 mice underwent bilateral renal ischemia–reperfusion (I/R) injury [46,47] for 30 min to induce ischemic AKI. Toxic AKI was induced by a single intraperitoneal (i.p.) injection of cisplatin 20 mg/kg (Sigma, St. Louis, MO, USA) [48]. Rhabdomyolysis was induced by the intramuscular injection of 50% glycerol into the caudal muscles of each thigh, with saline at 7.5 mL/kg as a control [49].

### 4.3. CAZ-AVI Overexposure-Based AKI Model

CAZ-AVI was administered subcutaneously (s.c.) per published murine pharmacology data [50,51,52]. A report demonstrated that 625 mg/kg s.c. corresponds to approximately 5 times the usual human dose as well as systemic and intestinal exposures comparable to those in human volunteers [53,54,55,56]. Thus, 625 mg/kg was defined as the mouse equivalent of high clinical exposure. To model the supratherapeutic conditions exceeding the maximum exposure observed in patients with renal dysfunction, a 2-fold higher dose (1250 mg/kg) was selected as the over-exposure (overdose) condition.

CAZ-AVI was dissolved in sterile phosphate-buffered saline (PBS) at a 4:1 ratio. Mice received s.c. injections, once daily for 3 consecutive days (days 0–2) at 0.10–0.15 mL per mouse to ensure consistent absorption. Control animals received an equal volume of PBS. All injections were administered into the dorsal s.c. space with a 29-gauge needle.

In addition, the dose selection was guided by pharmacokinetic considerations and established murine precedents. The clinically relevant dose (125 mg/kg/day) corresponds to the approximate mg/kg equivalent of the standard human CAZ-AVI regimen. The high-exposure dose (625 mg/kg/day) follows the precedent established by Hausman et al. [55], who demonstrated that five times the usual human dose is required to achieve consistently detectable antibiotic concentrations in mice. The supratherapeutic dose (1250 mg/kg/day) was selected to model markedly elevated exposure analogous to the worst-case accumulation observed in patients with renal dysfunction or delayed dose adjustment. Because direct LC–MS/MS quantification of ceftazidime and avibactam is technically challenging, functional PK surrogate assays (β-lactamase inhibition for avibactam and antibacterial activity for ceftazidime) were used to confirm graded functional exposure across the three dose levels.

The use of the s.c. route is also supported by methodological considerations. Repeated intravenous administration in mice can cause vein irritation, local inflammation, inconsistent dosing, and stress-related physiological changes that may confound renal injury assessment. In contrast, s.c. administration provides reproducible dosing with minimal vascular stress and is widely used for repeated high-dose antibiotic delivery in murine studies, including the CAZ-AVI protocol described by Hausman et al. [55] Adopting this validated s.c. regimen ensured methodological consistency and minimized procedural variability in our AKI model.

### 4.4. ATM-AVI Overexposure AKI Model

To evaluate whether supratherapeutic exposure to aztreonam–avibactam (ATM-AVI) can induce AKI, we designed a dosing regimen based on murine pharmacokinetic data [57,58,59]. Subcutaneous administration of ATM at 100 mg/kg and AVI at 100 mg/kg once daily was defined as the clinical-equivalent exposure, corresponding to drug levels achieved with recommended human doses [60,61,62,63]. An overdose condition was then established by administering a 2-fold greater dose: ATM at 200 mg/kg plus AVI at 200 mg/kg. Mice received s.c. injections into the dorsal region once daily for 3 consecutive days. Renal function and histology were assessed 48 h after the final dose; they demonstrated that the overdose group had elevated blood urea nitrogen (BUN) and serum creatinine, accompanied by PT injury comparable to that observed in established murine AKI models. These findings confirmed that high-level ATM-AVI exposure can trigger AKI in vivo.

### 4.5. RNA Sequencing (RNA-Seq)

RNA-seq was used to compare gene expression profiles in the kidneys of 8-week-old CKO and control mice. The accession numbers for raw and processed data will be deposited in the Gene Expression Omnibus repository upon acceptance of the paper for publication.

### 4.6. RNA Isolation, Reverse Transcription, and PCR

RNA processing, isolation, and PCR were carried out per an established protocol along with the same instruments and products mentioned [46]. However, primers were selected to meet the specific requirements of this study (Table 2).

### 4.7. Statistical Analysis

All statistical analyses were conducted using GraphPad Prism 8 (GraphPad Software, San Diego, CA, USA). Sample size was determined based on power calculations to ensure statistical robustness while adhering to the three reductions (3R) principle. Data are presented as the mean ± standard error, and groups were compared.

## Figures and Tables

**Table 1:** Mice used in the experiments and their breeding protocol.

Offspring	Breeding Process
Heterozygous transgenic	PT-specific *Gusb* transgenic mice (C57BL/6J) crossed with WT C57BL/6 mice
PT-specific *Gusb* knockout (Gusb^−/−^) mice (CKO mice)	*Gusb* flox/flox mice (C57BL/6J) crossed with γGT-Cre mice
Three types of control mice	Genotypes created during breeding: *Gusb* flox/flox *Gusb* flox/+ *Gusb* flox/+/γGT-Cre transgenic/+
*Gusb* flox/flox/γGT-Cre transgenic/+ (PT-specific Gusb^−/−^) mice (*Gusb* CKO mice)	*Gusb* flox/+/γGT-Cre transgenic/+ mice crossed with *Gusb* flox/flox mice

*Gusb*, β-glucuronidase; CKO, conditional knockout; PT, proximal tubule; WT, wild-type.

**Table 2:** Primers used to PCR_amplify the target genes.

Name	Forward	Reverse
*Gusb*	F: 5′- TGCTGTGAGGAGGAGGACTA-3′	R(5′-CAGCAGTTGGTGATGGTGTT -3′)
*Ccl2* (MCP-1)	F(5′-AGCTGTAGTTCTCAGCCGCT-3′)	R(5′-TCTGGACCACTTCTGCTTGG-3′)
*C3*	F(5′-GGAGCTGCTGACTTCTACCT-3′)	R(5′-TGCTTCAGGTCAGGTAGGTT-3′)
*Lcn2* (NGAL)	F(5′-CCTGGAGAACTGTGGTGCTA-3′)	R(5′-GCAGTCTCCTTCAGTGGTCA-3′)
*Slc7a13* (AGT1)	F(5′-TGCCTGGACTTCTTCTTCAC-3′)	R(5′-AGGTGAGGAGGAGAAGGAGA-3′)
*Acsm3*	F(5′-GACCTGGAGAAGATGGAGGA-3′)	R(5′-CTCCTGGTTGTCCTTGGTCT-3′)
*Aqp8*	F(5′-CAGGAGGAGTTCTTCCTGGA-3′)	R(5′-GGTAGCAGGTCAGGAGTTGA-3′)
*Gapdh*	F(5′-CCAGGGCTGCTTTTAACTC-3′)	R(5′-GCTCCCCCCTGCAAATGA-3′)

*Gusb*, β-glucuronidase; *Ccl2*, C-C motif chemokine ligand 2 (MCP-1); *C3*, complement component 3; *Lcn2*, lipocalin-2 (NGAL); *Slc7a13*, solute carrier family 7 member 13 (AGT1); *Acsm3*, acyl-CoA synthetase medium-chain family member 3; *Aqp8*, aquaporin-8; *Gapdh*, glyceraldehyde-3-phosphate dehydrogenase.

**Table 3:** Genes upregulated in the kidneys of CKO animals compared with control (Cont) mice.

Transcript_ID	Gene_Symbol	Description	CKO/Control.fc	CKO/Control.raw.pval
NM_009252	*Serpina3n*	Serine (or cysteine) peptidase inhibitor, clade A, member 3N	**215.809297**	**1.20939 × 10^−71^**
NM_008491	*Lcn2*	Lipocalin 2	**163.964302**	**7.2806 × 10^−121^**
NM_001039537	*Lif*	Leukemia inhibitory factor	**101.414973**	**8.55369 × 10^−61^**
NM_013701	*Ugt1a2*	UDP glucuronosyltransferase 1 family, polypeptide A2	**95.630069**	**5.1227 × 10^−109^**
NM_013468	*Ankrd1*	Ankyrin repeat domain 1 (cardiac muscle)	**85.460462**	**6.17471 × 10^−78^**
NM_023256	*Krt20*	Keratin 20	**83.941040**	**9.60475 × 10^−59^**
NM_008176	*Cxcl1*	Chemokine (C-X-C motif) ligand 1	**80.316550**	**7.97239 × 10^−74^**
NM_009778	*C3*	Complement component 3	**79.523367**	**3.3227 × 10^−132^**
NM_001301295	*Cidec*	Cell death-inducing DFFA-like effector c	**77.938875**	**2.62332 × 10^−56^**
NM_080457	*Muc4*	Mucin 4	**75.341768**	**5.27599 × 10^−51^**
NM_011333	*Ccl2*	Chemokine (C-C motif) ligand 2	**61.632396**	**3.8486 × 10^−73^**
NM_001301404	*Serpina10*	Serine peptidase inhibitor, clade A member 10	**57.132938**	**7.80895 × 10^−81^**
NM_018827	*Crlf1*	Cytokine receptor-like factor 1	**44.525429**	**1.1458 × 10^−31^**
NM_001329965	*Hp*	Haptoglobin	**44.368733**	**1.46562 × 10^−31^**
NM_001163525	*S100a14*	S100 calcium-binding protein A14	**43.189499**	**5.52522 × 10^−39^**
NM_001077499	*Scn8a*	Sodium channel, voltage-gated, type VIII, alpha	**41.980834**	**1.29939 × 10^−19^**
NM_009912	*Ccr1*	Chemokine (C-C motif) receptor 1	**41.694694**	**2.61638 × 10^−25^**
NM_011448	*Sox9*	SRY (sex determining region Y)-box 9	**40.030465**	**8.01079 × 10^−37^**
NM_007822	*Cyp4a14*	Cytochrome P450, family 4, subfamily a, polypeptide 14	**38.075113**	**7.81142 × 10^−94^**
NM_013820	*Hk2*	Hexokinase 2	**32.948498**	**3.74081 × 10^−42^**
NM_001313963	*Krt19*	Keratin 19	**32.317442**	**2.01591 × 10^−68^**
NM_030601	*Clca3a2*	Chloride channel accessory 3A2	**31.194596**	**4.86258 × 10^−42^**
NM_007498	*Atf3*	Activating transcription factor 3	**30.239338**	**9.68825 × 10^−59^**
NM_00116162	*Aoc1*	Amine oxidase, copper-containing 1	**29.880417**	**7.09572 × 10^−81^**
NM_001290825	*Col17a1*	Collagen, type XVII, alpha 1	**29.482644**	**3.3081 × 10^−23^**
NM_009140	*Cxcl2*	Chemokine (C-X-C motif) ligand 2	**29.465216**	**2.12555 × 10^−19^**
NM_001370743	*Ltbp2*	Latent transforming growth factor beta binding protein 2	**28.628857**	**9.81377 × 10^−46^**
NM_023544	*Rsc1a1*	Regulatory solute carrier protein, family 1, member 1	**27.815748**	**2.18538 × 10^−58^**
NM_001355020	*Cnr1*	Cannabinoid receptor 1 (brain)	**27.288548**	**6.70597 × 10^−14^**
NM_053138	*Pcdhb13*	Protocadherin beta 13	**26.995422**	**2.11169 × 10^−08^**
NM_001039150	*Cd44*	CD44 antigen	**26.625232**	**1.79939 × 10^−61^**
NM_001347458	*Egr2*	Early growth response 2	**26.208123**	**1.32407 × 10^−35^**
NM_001170332	*Clec4a2*	C-type lectin domain family 4, member a2	**24.958764**	**8.6943 × 10^−23^**
NM_007707	*Socs3*	Suppressor of cytokine signaling 3	**23.123902**	**3.18626 × 10^−59^**
NM_026835	*Ms4a6d*	Membrane-spanning 4-domains, subfamily A, member 6D	**23.028700**	**3.36874 × 10^−32^**
NM_172604	*Scara3*	Scavenger receptor class A, member 3	**22.431338**	**4.71029 × 10^−18^**
NM_001313760	*Tlr8*	Toll-like receptor 8	**22.339873**	**1.81338 × 10^−23^**
NM_011426	*Siglec1*	Sialic acid-binding Ig-like lectin 1, sialoadhesin	**22.233107**	**1.82712 × 10^−50^**
NM_001204201	*Spp1*	Secreted phosphoprotein 1	**22.199729**	**3.95442 × 10^−80^**
NM_001166376	*Ms4a6c*	Membrane-spanning 4-domains, subfamily A, member 6C	**21.495620**	**1.22494 × 10^−47^**
NM_177355	*Plcxd3*	Phosphatidylinositol-specific phospholipase C, X domain containing 3	**21.166762**	**1.01667 × 10^−10^**
NM_009181	*St8sia2*	ST8 alpha-N-acetyl-neuraminide alpha-2,8-sialyltransferase 2	**20.248494**	**2.47547 × 10^−10^**
NM_001356515	*Sult4a1*	Sulfotransferase family 4A, member 1	**20.080903**	**2.94037 × 10^−16^**
NM_001190320	*Clec4n*	C-type lectin domain family 4, member n	**20.080903**	**2.94037 × 10** **^−^** **^16^**
NM_008871	*Serpine1*	Serine (or cysteine) peptidase inhibitor, clade E, member 1	**19.441880**	**1.63163 × 10** **^−^** **^47^**
NR_151608	*Gm34746*	Predicted gene, 34,746	**19.391448**	**3.61809 × 10** **^−^** **^06^**
NM_199223	*Rtn4rl2*	Reticulon 4 receptor-like 2	**18.827338**	**7.36057 × 10** **^−^** **^16^**

**Table 4:** Genes downregulated in the kidneys of CKO animals compared with control (Cont) mice.

Transcript_ID	Gene_Symbol	Description	CKO/Control. fc	CKO/Control.raw.pval
NM_001163032	*Synpr*	Synaptoporin	**−184.752401**	**4.56864 × 10^−93^**
NM_001077425	*Cntnap5a*	Contactin-associated protein-like 5A	**−63.317877**	**4.86997 × 10^−57^**
NM_144856	*Slc22a7*	Solute carrier family 22 (organic anion transporter), member 7	**−46.969746**	**1.92086 × 10^−97^**
NM_007733	*Col19a1*	Collagen, type XIX, alpha 1	**−37.256227**	**2.32349 × 10^−34^**
NR_105025	*D14Ertd670e*	DNA segment, Chr 14, ERATO Doi 670, expressed	**−36.251305**	**2.75344 × 10^−15^**
NM_021408	*Ush2a*	Usherin	**−35.410878**	**2.17202 × 10^−38^**
NM_001081688	*Tmprss9*	Transmembrane protease, serine 9	**−34.320377**	**8.05883 × 10^−54^**
NM_016870	*Acsm3*	Acyl-CoA synthetase medium-chain family member 3	**−32.721222**	**1.54157 × 10^−95^**
NM_053176	*Hrg*	Histidine-rich glycoprotein	**−30.417454**	**5.62244 × 10^−21^**
NM_013797	*Slco1a1*	Solute carrier organic anion transporter family, member 1a1	**−26.895058**	**1.52304 × 10^−87^**
NM_010594	*Kap*	Kidney androgen-regulated protein	**−19.905802**	**7.25348 × 10^−76^**
NM_145539	*Tm4sf4*	Transmembrane 4 superfamily member 4	**−17.930626**	**4.45768 × 10^−38^**
NM_009349	*Inmt*	Indolethylamine N-methyltransferase	**−16.622394**	**1.78181 × 10^−68^**
NM_001359131	*Mpped1*	Metallophosphoesterase domain containing 1	**−16.078220**	**2.14524 × 10^−51^**
NM_001013411	*Nkain2*	Na^+^/K^+^ transporting ATPase interacting 2	**−14.870007**	**1.18072 × 10^−08^**
NM_001102607	*Col6a6*	Collagen, type VI, alpha 6	**−14.503409**	**7.74976 × 10^−59^**
NM_001001320	*Tbx10*	T-box 10	**−13.806768**	**1.71995 × 10^−36^**
NM_007376	*Pzp*	PZP, alpha-2-macroglobulin-like	**−13.791309**	**3.468 × 10^−59^**
NM_001272045	*Ces2h*	Carboxylesterase 2H	**−13.656377**	**2.08081 × 10^−22^**
NM_177002	*Slc22a30*	Solute carrier family 22, member 30	**−13.124345**	**1.98326 × 10^−59^**
NM_001278271	*Gm44504*	Predicted readthrough transcript (NMD candidate), 44,504	**−13.054281**	**6.55924 × 10^−17^**
NM_198171,XM	*Ces2b*	Carboxyesterase 2B	**−12.828360**	**2.43128 × 10^−46^**
NM_001109045	*Aqp8*	Aquaporin 8	**−12.215185**	**1.99436 × 10^−12^**
NM_001347022	*Snx31*	Sorting nexin 31	**−12.168022**	**1.06307 × 10^−47^**
NM_030689	*Nptxr*	Neuronal pentraxin receptor	**−12.120500**	**1.69869 × 10^−09^**
NM_007825	*Cyp7b1*	Cytochrome P450, family 7, subfamily b, polypeptide 1	**−11.682095**	**1.39891 × 10^−53^**
NM_053188	*Srd5a2*	Steroid 5 alpha-reductase 2	**−11.611276**	**8.5532 × 10^−45^**
NM_001357025	*Gusb*	Glucuronidase, beta	**−11.582189**	**7.11998 × 10^−55^**
NM_001278569	*Cryaa*	Crystallin, alpha A	**−11.213679**	**7.06058 × 10^−09^**
NM_001081643	*Xlr3b*	X-linked lymphocyte-regulated 3B	**−11.213679**	**7.06058 × 10^−09^**
NM_145530	*Rhov*	Ras homolog family member V	**−11.213245**	**1.67668 × 10^−33^**
NM_011915	*Wif1*	Wnt inhibitory factor 1	**−10.913738**	**1.61919 × 10^−07^**
NM_001034872	*Ldc1*	Leucine decarboxylase 1	**−10.828637**	**2.99784 × 10^−49^**
NM_001013820	*Slc22a28*	Solute carrier family 22, member 28	**−10.497155**	**3.97824 × 10^−51^**
NM_001160402	*Tm4sf19*	Transmembrane 4 L six family member 19	**−10.423494**	**1.7433 × 10^−05^**
NM_020279	*Ccl28*	Chemokine (C-C motif) ligand 28	**−10.410239**	**4.30518 × 10^−47^**
NM_030174	*Mctp1*	Multiple C2 domains, transmembrane 1	**−10.283563**	**6.95177 × 10^−25^**
NM_001039554	*Angptl7*	Angiopoietin-like 7	**−10.099244**	**4.20241 × 10^−50^**
NM_028746	*Slc7a13*	Solute carrier family 7, member 13	**−9.890109**	**2.61953 × 10** **^−^** **^49^**
NM_001040699	*Mtmr7*	Myotubularin-related protein 7	**−9.780824**	**2.07441 × 10** **^−^** **^34^**
NM_013697	*Ttr*	Transthyretin	**−9.434765**	**5.3381 × 10** **^−^** **^43^**
NM_013614	*Odc1*	Ornithine decarboxylase, structural 1	**−9.310815**	**2.59977 × 10** **^−^** **^47^**
NM_00102993	*Sec14l3*	SEC14-like lipid binding 3	**−9.270784**	**6.53248 × 10** **^−^** **^37^**
NM_001134697	*Ctxn3*	Cortexin 3	**−9.270265**	**1.07257 × 10** **^−^** **^16^**
NM_008719	*Npas2*	Neuronal PAS domain protein 2	**−9.071487**	**1.44298 × 10** **^−^** **^31^**
NM_146052	*Lrrc3b*	Leucine-rich repeat containing 3B	**−9.038380**	**3.61809 × 10** **^−^** **^06^**
NM_176920	*Lrtm1*	Leucine-rich repeats and transmembrane domains 1	**−9.038380**	**3.61809 × 10** **^−^** **^06^**
NM_134072	*Akr1c14*	Aldo-keto reductase family 1, member C14	**−9.030561**	**4.98059 × 10** **^−^** **^46^**

## Data Availability

The RNA-seq datasets generated and/or analyzed in this study—proximal tubule-specific Gusb conditional knockout (CKO) versus control (PT-Gusb CKO vs. Control)—are openly available in the Gene Expression Omnibus (GEO) under accession number GSE335589. The data can be accessed at https://www.ncbi.nlm.nih.gov/geo/query/acc.cgi?acc=GSE335589 (accessed on 29 July 2026). Further information and requests for resources and reagents are available from the corresponding author (kazuhiro@tokushima-u.ac.jp).

## Supplementary Materials

The following supporting information can be downloaded at: https://www.mdpi.com/article/10.3390/ijms27209045/s1.
