# Supplementary material for: Proximal Tubule-to-Proximal Tubule Efferocytosis: Gusb Prevents Efferocytosis Failure and Polyphagolysosomal Overload in CAZ-AVI-Induced Acute Kidney Injury
Source: Int J Mol Sci. 2026 Oct 12;27(20):9045. doi: 10.3390/ijms27209045 (PMC13705548; doi:10.3390/ijms27209045)
Supplement: Supplementary file 1 [file ijms-27-09045-s001.zip › ijms-4501355-supplementary.pdf]

Supplemental Material Table of Contents

Supplementary Methods

Supplemental Figure S1. Kyoto Encyclopedia of Genes and Genomes (KEGG) pathways identified from RNA sequencing analysis, showing the efferocytosis pathway *via* a comparison of CKO and control mice.

Supplemental Figure S2. KEGG pathways recognized from RNA sequencing analysis, demonstrating the glutathione pathway *via* a comparison of CKO and control mice.

Supplemental Figure S3. KEGG pathways enriched from RNA sequencing analysis, revealing the lysosome pathway *via* a comparison of CKO and control mice.

Supplementary Figure S4. Dose-dependent CAZ-AVI–induced acute kidney injury in mice, including BUN/creatinine changes and PAS-based tubular injury scores.

Supplementary Figure S5. Genotyping of Gusb floxed, γ-GT–Cre, and proximal tubule–specific Gusb CKO mice using PCR.

Supplementary Figure S6. Plasma albumin levels and glomerular structural markers (Nephrin, Podocin) in Gusb CKO mice.

Supplementary Figure S7. Identification of apoptotic proximal tubule cells in CAZ-AVI–induced AKI using three proximal tubule markers (AQP-1, LTL, Megalin).

Supplementary Figure S8. Lineage-traced proximal tubule cells undergoing apoptosis in CAZ-AVI–induced AKI (tdTomato × TUNEL).

Supplementary Methods

Transgenic and CKO Mice

Murine Gusb cDNA (GenBank accession number: NM_010368.1) containing the full-length

open reading frame was cloned. To construct the expression vector and generate a renal PT-specific transgenic mouse, the murine Gusb cDNA sequence was ligated to the mouse sodium phosphate cotransporter IIa (Npt2) promoter. This clone was designated Npt2-Gusb. Mice were propagated as heterozygous transgenic types by breeding them with wild-type (WT) C57BL/6 mice. F_2 mice were considered transgenics, with age- and sex-matched littermates serving as WT controls.

To generate Gusb loxP/loxP mice, the targeting vector was constructed by inserting a self-deleting anchor-flanked neomycin cassette and two loxP sites flanking exons 1–2 of Gusb. These were then inserted by electroporating embryonic stem cells from C57BL/6J mice. After one subsequent cross with B6 animals, the neotransgene was excised, and the Gusb loxP/+ mice obtained were intercrossed to generate the Gusb loxP/loxP mice. These were crossed with Cag-CreER mice (Jackson Laboratory, 004682) to generate systemic Gusb knockout mice (Gusb loxP/loxP/Cag-$Cre^{+/-}$, $Gusb^{-/-}$); homozygous mice with Cag-Cre-negative littermates serving as the controls. For gene knockout, tamoxifen suspended in corn oil was administered intraperitoneally at 10 mg/mL and 100 mg/kg once every 24 h for 5 consecutive days. After 30 days of the last tamoxifen injection, tissues were harvested for analysis. Renal PT-specific *Gusb* knockout ($Gusb^{-/-}$) mice (CKO mice) were generated by crossing *Gusb* flox/flox mice having a C57BL/6J background with γGT-Cre mice (Jackson Laboratory, 012841). The following types of control mice were created: *Gusb* flox/flox, *Gusb* flox/+, and *Gusb* flox/+/γGT-Cre transgenic/+. In subsequent studies, *Gusb* flox/+/γGT-Cre transgenic/+ mice were crossed with *Gusb* flox/flox mice to generate *Gusb* flox/flox/γGT-Cre transgenic/+ (PT-specific $Gusb^{-/-}$) mice (*Gusb* CKO mice); *Gusb* flox/flox mice served as controls. Animal experiments were approved by the Tokushima University Animal

Committee (protocol number T2025–41, approval date 1 April 2025) and were conducted in accordance with institutional guidelines and the ARRIVE 2.0 recommendations.

RNA sequencing

RNA sequencing was used to compare the gene expression profiles in the kidneys of 8-week-old CKO and control mice. RNA was extracted from the left kidney following the protocol employed for quantitative PCR. Libraries were prepared using the TruSeq Stranded mRNA Kit (Illumina, San Diego, CA, USA) and sequenced on a NovaSeq sequencer (Illumina), resulting in an average read length of 100 bp.

STAR aligner (version 2.7.3a) was used for sequence alignment; HTSeq for raw read counting; DESeq2 for normalization; Cufflinks (version 2.2.1) for estimating gene expression levels, which were reported as fragments per kilobase of transcript per million mapped reads (FPKM) (https://www.bioinformatics.babraham.ac.uk/projects/fastqc/); and FastQC for data quality assessment, excluding low-quality reads from further analysis. Overrepresentation, gene set enrichment, and pathway analyses were conducted to identify significant variations in the expression levels of protein-encoding genes.

Serum metabolite quantification

Serum creatinine and blood urea nitrogen levels were quantified (mg/dL) using an automated chemistry analyzer. Albuminuria was quantified *via* SDS-PAGE.

Electron microscopy

After mouse kidney specimens were embedded in Epon epoxy resin (Hexion, Columbus, OH, USA), electron micrographs of ten proximal tubular cells per kidney were randomly selected for morphometric evaluation.

Immunofluorescence staining

Dual-label immunofluorescence staining was carried out using primary and secondary antibodies after overnight incubation of 5 µm cryostat kidney sections with the antibody mix. The primary antibodies included rabbit polyclonal anti-Gusb (ab16690: Abcam, Cambridge, UK), Odc1 (ab193338: Abcam), 4-HNE (HNEJ-2: Japan Institute for the control of Aging, Shizuoka, Japan), aquaporin-1 (AQP-1; B-11, Santa Cruz Biotechnology, Santa Cruz, CA, USA), and biotinylated lotus tetragonolobus lectin antibody (LTL) (L-132: Vector Laboratories), with secondary antibodies from Jackson ImmunoResearch Laboratories (West Grove, PA, USA). Double immunofluorescence staining for TUNEL and AQP-1 within kidneys was also used to localize CAZ-AVI-induced or Gusb defect-induced apoptosis.

Controls for the immunofluorescence experiments

Positive and negative controls were used to validate each primary antibody *via* immunofluorescence. As a positive control, kidneys from 8-week-old healthy WT mice (i.e., kidneys that express each target molecule) were harvested and used for immunofluorescence assays. For 4-HNE immunofluorescence, kidneys from 15-month-old WT mice were used, as aging kidneys demonstrate positive 4-HNE staining. Tests for the negative control employed immune depletion. For the negative control, the primary antibodies were blocked by incubation

with 5 mg/mL antigen solution at a 1:2 (v:v) ratio and 4°C overnight. Subsequently, the preblocked antibody was used as the primary antibody for the immunofluorescence assays.

TUNEL control assay

Each experiment included negative and positive controls. A labeled solution rather than the TUNEL reaction mix was used as the negative control. Mice's thymus with apoptotic thymocytes was used as the positive control.

CAZ-AVI overexposure model and assessment of acute kidney injury

To evaluate dose-dependent CAZ-AVI–induced acute kidney injury, eight-week-old male C57BL/6 mice were administered ceftazidime–avibactam (CAZ-AVI) subcutaneously at 125, 625, or 1,250 mg/kg/day for three consecutive days. CAZ-AVI was freshly dissolved in sterile PBS immediately before injection. Control mice received an equivalent volume of PBS. Forty-eight hours after the final injection, mice were anesthetized, and blood samples were collected via cardiac puncture. Serum blood urea nitrogen (BUN) and creatinine concentrations were quantified using an automated chemistry analyzer according to the manufacturer's instructions. Kidneys were harvested, fixed in 4% paraformaldehyde, dehydrated, and embedded in paraffin. Periodic acid–Schiff (PAS) staining was performed on 4-μm sections to assess tubular injury. Lysed proximal tubules were identified based on loss of brush border integrity, cytoplasmic dissolution, and luminal debris. Tubular injury scores were assigned in a blinded manner using a semiquantitative scoring system that evaluated the percentage of tubules exhibiting epithelial lysis or denudation.

Genotyping of Gusb floxed mice and γ-GT–Cre transgenic mice

Genomic DNA was extracted from clipped tail tissues using standard proteinase K digestion followed by ethanol precipitation. PCR-based genotyping was performed to identify wild-type, floxed, and Cre-positive alleles.

Gusb floxed allele genotyping

PCR amplification of the Gusb locus was performed using the following primer pair:

- Forward: ATAGGGACATCTCTGCTTCAGTTTA
- Reverse: CATGCTGGACACCAAGCTTTC

This primer set generated:

- 82 bp band → wild-type allele
- 133 bp band → floxed allele

Genotypes were interpreted as follows:

- 82 bp only: wild-type
- 82 bp + 133 bp: heterozygous floxed (fl/+)
- 133 bp only: homozygous floxed (fl/fl)

γ-GT–Cre transgene genotyping

PCR amplification of the γ-GT–Cre transgene was performed using:

- Forward: AGGTGTAGAGAAGGCACTTAGC
- Reverse: CTAATCGCCATCTTCCAGCAGG

This primer pair generated a 411-bp fragment, indicating the presence of the γ-GT–Cre transgene. Genotypes were classified as Cre/+ (positive) or +/+ (negative).

PCR reactions were performed using a routine protocol with Taq DNA polymerase under standard cycling conditions (initial denaturation at 94 °C, followed by 35 cycles of denaturation, annealing,

and extension). PCR products were separated on 2% agarose gels containing ethidium bromide and visualized under UV illumination.

To generate proximal tubule–specific Gusb conditional knockout (Gusb CKO) mice, Gusb flox/flox mice were crossed with γ-GT–Cre mice. Offspring were genotyped using the PCR assays described above. The fl/fl; Cre/+ genotype was designated as PT-specific Gusb knockout (Gusb CKO), whereas fl/fl mice served as littermate controls.

Plasma albumin quantification

Plasma albumin concentrations were measured using the same colorimetric assay kit employed for urinary albumin quantification (Shibayagi, Gunma, Japan), following the manufacturer's instructions. Blood samples were collected from 8-week-old control and Gusb CKO mice via cardiac puncture under anesthesia. Plasma was separated by centrifugation at 3,000 × g for 10 min at 4 °C. Albumin concentrations (g/L) were determined by spectrophotometric measurement at 620 nm, using a standard curve generated from purified mouse albumin. All measurements were performed in duplicate.

Immunofluorescence staining for Nephrin and Podocin

Kidneys were harvested from 8-week-old control and Gusb CKO mice, embedded in OCT compound, and sectioned at 5 µm thickness using a cryostat. Sections were air-dried and rehydrated in PBS, followed by permeabilization with 0.1% Triton X-100 for 10 min. For Nephrin staining, sections were incubated overnight at 4 °C with guinea pig anti-Nephrin antibody (1:50; Progen, Heidelberg, Germany). For Podocin staining, sections were incubated with FITC-conjugated polyclonal Podocin antibody (Cat. No. bs-6597R-FITC; 1:200; Bioss

Antibodies). After primary antibody incubation, sections were washed twice in PBST (PBS + 0.05% Tween-20) for 10 min each. Nephrin-stained sections were incubated with species-appropriate secondary antibodies (Jackson ImmunoResearch Laboratories, West Grove, PA, USA) for 1 h at room temperature. All sections were mounted using a DAPI-containing mounting medium (Vector Laboratories). Fluorescence images were acquired in a blinded and unbiased manner using a fluorescence microscope (Olympus BX53) equipped with MagnaFire Digital Firewire Camera Software. A violet filter was used for DAPI and a green filter for FITC.

Immunofluorescence staining for proximal tubule markers and TUNEL

Kidneys were harvested from PBS-treated control mice and CAZ-AVI–treated mice (1,250 mg/kg/day, subcutaneous, for three consecutive days). Tissues were embedded in OCT compound and sectioned at 5 µm thickness using a cryostat. Sections were air-dried, rehydrated in PBS, and permeabilized with 0.1% Triton X-100 for 10 min.

Dual-label immunofluorescence staining was performed after overnight incubation of sections at 4 °C with primary antibodies. The following proximal tubule markers were used:

- Aquaporin-1 (AQP1): rabbit anti-AQP1 (B-11; Santa Cruz Biotechnology; 1:50)
- Lotus tetragonolobus lectin (LTL): biotinylated LTL (L-132; Vector Laboratories; 1:200)
- Megalin (LRP2): rabbit anti-Megalin (Abcam, ab76969; 1:50)

For apoptosis detection, TUNEL staining was performed using a commercial TUNEL reaction kit according to the manufacturer’s protocol. TUNEL-positive nuclei were visualized in red, whereas proximal tubule markers were visualized in green.

After primary antibody incubation, sections were washed twice in PBST (PBS + 0.05% Tween-20) for 10 min each. For LTL staining, streptavidin-conjugated fluorophores (Jackson

ImmunoResearch Laboratories) were applied for 1 h at room temperature. All sections were mounted using a DAPI-containing mounting medium (Vector Laboratories).

Fluorescence images were acquired in a blinded and unbiased manner using a fluorescence microscope (Olympus BX53) equipped with MagnaFire Digital Firewire Camera Software. A violet filter was used for DAPI and a green filter for FITC or Alexa Fluor-conjugated secondary antibodies.

Quantification of apoptotic proximal tubule cells

For each mouse, ≥10 fields were randomly selected. The number of TUNEL-positive cells within AQP1-positive, LTL-positive, or Megalin-positive proximal tubules was counted manually by an observer blinded to group allocation. The density of apoptotic cells was expressed as TUNEL-positive cells per mm^2. All quantitative analyses were performed using $n = 7$ mice per group, and data were expressed as means ± SEM. Statistical comparisons were conducted using a two-tailed Student's t-test, and exact P-values are reported in the corresponding figure panels.

Generation of SLC34a1-GCE; Rosa26-tdTomato lineage-tracing mice

Proximal tubule lineage-tracing mice were generated by crossing SLC34a1-GCE knock-in mice (Jackson Laboratory, Stock No. 032285) with Rosa26-tdTomato reporter mice (Ai14). The Ai14 reporter line carries the Gt(ROSA)26Sor^tm14(CAG-tdTomato)Hze allele, a Cre-dependent tdTomato reporter widely used for lineage-tracing studies (Jackson Laboratory, Stock No. 007914). In this system, Cre-mediated recombination activates robust tdTomato expression under the CAG promoter following excision of a loxP-flanked STOP cassette.

The SLC34a1-GCE allele contains an eGFPCreERT2 (GCE) transgene inserted at the initiation codon of the *Slc34a1* locus, enabling tamoxifen-inducible Cre recombination specifically in proximal tubule epithelial cells. The GCE transgene comprises an enhanced green fluorescent protein (eGFP) fused to a tamoxifen-inducible Cre recombinase (CreERT2). This knock-in allele was originally generated by homologous recombination in embryonic stem cells using a targeting construct containing:

- a frt-flanked PGK-neo selection cassette,
- an MC1-TK negative selection cassette,
- 2.6-kb 5′ and 4.5-kb 3′ homology arms flanking the insertion site.

Positive ES cell clones were identified by long-range PCR and used for blastocyst injection to establish the mouse line.

Tamoxifen-induced proximal tubule lineage labeling

Eight-week-old SLC34a1-GCE; Rosa26-tdTomato mice received tamoxifen (10 mg/mL in 3% ethanol/corn oil) via intraperitoneal injection every other day for five doses to induce proximal tubule-specific CreERT2 activation. This regimen resulted in robust tdTomato expression in proximal tubule epithelial cells. Mice were allowed to recover for 5 days after the final tamoxifen injection before CAZ-AVI exposure.

CAZ-AVI–induced acute kidney injury

Following lineage labeling, mice received either PBS (control) or CAZ-AVI at 1,250 mg/kg/day, administered subcutaneously for three consecutive days. Kidneys were harvested 48 h after the final injection.

Triple immunofluorescence staining for tdTomato, TUNEL, and DAPI

Kidneys were embedded in OCT compound and sectioned at 5 μm thickness. Sections were rehydrated in PBS and permeabilized with 0.1% Triton X-100 for 10 min.

Apoptotic cells were detected using a commercial TUNEL reaction kit according to the manufacturer's protocol. tdTomato fluorescence was detected directly without antibody amplification. Nuclei were counterstained with DAPI (Vector Laboratories).

Sections were washed twice in PBST (PBS + 0.05% Tween-20) and mounted with DAPI-containing medium.

Fluorescence images were acquired in a blinded and unbiased manner using an Olympus BX53 fluorescence microscope equipped with MagnaFire Digital Firewire Camera Software. A violet filter was used for DAPI and a red filter for tdTomato/TUNEL.

Quantification of lineage-traced apoptotic proximal tubule cells

For each mouse, ≥10 tdTomato-positive tubules were randomly selected. The **apoptosis index** was calculated as:

$$\text{Apoptosis Index} = \frac{\text{Number of TUNEL-positive cells within tdTomato-positive proximal tubules}}{\text{Total number of tdTomato-positive proximal tubule cells}}$$

Cell counting was performed manually by an observer blinded to group allocation. All quantitative analyses were performed using n = 7 mice per group, expressed as means ± SEM, and analyzed using a two-tailed Student's t-test. Exact P-values are reported in the corresponding figure panels.

ARRIVE 2.0–Compliant Reporting of Animal Experiments

In accordance with the ARRIVE 2.0 Essential 10 and Recommended Set, we provide the following clarifications regarding study design, sample size determination, randomization, blinding, animal care, and statistical analysis.

Study design and experimental unit

All experiments were conducted using 8-week-old male C57BL/6 mice. Experimental groups included WT, Gusb-CKO, Gusb-Tg, saline-treated controls, CAZ-AVI dosing groups (125, 625, and 1,250 mg/kg/day), and ATM-AVI dosing groups (100/100 and 200/200 mg/kg/day). The experimental unit was the individual mouse.

Sample size and justification

Sample size was determined by power calculations designed to detect differences in the primary outcome (Odc1 expression) while adhering to the 3R principle. Exact n values for each experiment are reported in the figure legends.

Inclusion/exclusion criteria and attrition

No animals or data points were excluded. No attrition occurred during the study. All animals that entered the study were included in the final analyses.

Randomization and confounders

Randomization was not used because group allocation was determined by genotype (WT, Gusb-CKO, Gusb-Tg) or treatment protocol (saline, CAZ-AVI, ATM-AVI). Cage location and treatment order were not explicitly randomized; however, all animals were housed under identical environmental conditions and treated at consistent times.

Blinding

Blinding was not feasible because genotype-based allocation and treatment protocols were readily identifiable. All histological, molecular, and imaging analyses (PAS, TUNEL, immunostaining, TEM, PCR) were performed with investigators aware of group assignments.

Outcome measures

Outcome measures included renal function (BUN, creatinine), tubular injury (PAS), apoptosis (TUNEL), oxidative stress (4-HNE), ultrastructural features (TEM), albuminuria (SDS-PAGE), and transcriptomic changes (RNA-seq). The primary outcome was Odc1 expression in proximal tubules.

Animal care, monitoring, and ethics

All procedures were performed under standard laboratory conditions. Mice were monitored daily for general health, activity, and distress. No unexpected adverse events occurred. All animal experiments were approved by the Tokushima University Animal Experiment Committee (protocol T2025-41; approved April 1, 2025) and conducted in accordance with institutional guidelines and ARRIVE 2.0 recommendations.

Statistical analysis

Statistical analyses were performed using GraphPad Prism 8. Two-group comparisons used unpaired two-tailed Student's t-tests. Multi-group comparisons used one-way or two-way ANOVA with Tukey's post-hoc tests. Data are presented as mean ± SEM. RNA-seq differential expression analysis was performed using STAR, HTSeq, DESeq2, and Cufflinks, with significance defined as adjusted $P < 0.05$ and $|\log_2 FC| > 1$.

Reagents and Sources, RNA Sequencing Procedures, and Data Processing

All reagents used in this study were obtained from certified commercial suppliers, and their company names, cities, and countries are provided to ensure full reproducibility. CAZ-AVI was purchased from Merck (Darmstadt, Germany); aztreonam (ATM) and cisplatin were obtained from Sigma-Aldrich (St. Louis, MO, USA); avibactam (AVI) from MedChemExpress (Monmouth Junction, NJ, USA); glycerol from FUJIFILM Wako Pure Chemical Industries (Osaka, Japan); phosphate-buffered saline (PBS) from Gibco, Thermo Fisher Scientific (Waltham, MA, USA); and 29-gauge needles from Terumo Corporation (Tokyo, Japan). Reagents used for RNA extraction, reverse transcription, and PCR are described in Section 4.6 of the main text, with manufacturer details included therein.

RNA sequencing was performed to compare renal gene expression profiles between 8-week-old CKO and control mice. Total RNA was extracted from the left kidney using the same protocol applied for quantitative PCR. Sequencing libraries were prepared using the TruSeq Stranded mRNA Kit from Illumina (San Diego, CA, USA) and sequenced on an Illumina NovaSeq system (San Diego, CA, USA), generating paired-end reads with an average read length of 100 bp. Bioinformatic analyses followed established pipelines: STAR aligner (version 2.7.3a) was used for sequence alignment, HTSeq for raw read counting, and DESeq2 for normalization. Gene expression levels were estimated using Cufflinks (version 2.2.1) and reported as fragments per kilobase of transcript per million mapped reads (FPKM). Data quality was assessed using FastQC, and the FastQC documentation website was accessed on 10 October 2026. Overrepresentation, gene set enrichment, and pathway analyses were performed to identify significant changes in protein-coding gene expression. The complete dataset has been deposited in the Gene Expression Omnibus under accession number GSE335589 and is publicly available.

This supplementary section integrates all reagent information, software version numbers, and website access dates, thereby fully addressing the editorial requirements for transparency and reproducibility.

Supplementary Figure Legends

Supplementary Figures S1–3: Kyoto Encyclopedia of Genes and Genomes (KEGG) pathways identified from the RNA sequencing analysis by comparing conditional KO and control mice.

RNA-seq-related KEGG enrichment of downregulated differentially expressed genes (x-axis: gene ratio; y-axis: KEGG terms). The size of each circle represents the gene count. The color of each circle indicates the adjusted *P*-value. Efferocytosis (S-Figure S1), glutathione pathway (S-Figure S2), and lysosome (S-Figure S3) pathways are shown.

Supplementary Figure S4：Dose-dependent CAZ-AVI–induced acute kidney injury in mice.

(a) Experimental timeline of the CAZ-AVI overexposure model. Eight-week-old C57BL/6 mice received subcutaneous CAZ-AVI at 125, 625, or 1,250 mg/kg/day for three consecutive days. Serum blood urea nitrogen (BUN) (left) and creatinine (right) levels were measured 48 h after the final injection. (b) Representative Periodic acid–Schiff (PAS) staining of kidney sections obtained 48 h after CAZ-AVI exposure. Arrows indicate lysed proximal tubules. Quantitative tubular injury scores are shown on the right. All quantitative data in this figure were obtained from n = 7 mice per group and are presented as means ± SEM. Statistical analyses were performed

using one-way ANOVA with Bonferroni correction. Scale bars: 50 µm. Exact P-values are indicated in each panel.

Supplementary Figure S5 : Genotyping of Gusb floxed mice, γ-GT–Cre mice, and proximal tubule–specific Gusb conditional knockout (Gusb CKO) mice. (a) PCR genotyping of Gusb floxed alleles. Representative PCR results obtained from genomic DNA extracted from clipped tail tissues. The wild-type Gusb allele (82 bp) and the floxed Gusb allele (133 bp) are indicated. Genotypes of representative mice are shown. (b) PCR validation of γ-GT–Cre transgenic mice. PCR amplification of the γ-GT–Cre transgene (411 bp) is shown. Genotypes of representative litters (Cre/+ or +/+) are indicated. (c) Generation of proximal tubule–specific Gusb CKO mice. PCR genotyping of offspring obtained by crossing Gusb floxed mice with γ-GT–Cre mice. The floxed Gusb allele (133 bp) appears as a double band in heterozygous mice (fl/+) and a single band in homozygous floxed mice (fl/fl). Representative genotypes are indicated: fl/fl; Cre/+ = proximal tubule–specific Gusb CKO, fl/+; Cre/+ = heterozygous Gusb CKO.

Supplementary Figure S6. Plasma albumin levels and glomerular structural markers in Gusb CKO mice. (a) Plasma albumin levels. Plasma albumin concentrations (g/L) in control (Cont) and Gusb conditional knockout (CKO) mice at 8 weeks of age (n = 7 per group). Data are presented as means ± SEM and analyzed using a two tailed Student's t test. Exact P values are indicated. Plasma albumin levels were unchanged between groups, indicating the absence of systemic hypoalbuminemia. (b) Nephrin immunofluorescence. Representative confocal images of glomeruli stained for Nephrin (green) and DAPI (blue). Fluorescence images were acquired in a blinded and unbiased manner using a fluorescence microscope equipped with MagnaFire Digital

Firewire Camera Software. The Nephrin positive area relative to total glomerular area was quantified using Image Pro Plus Image Analysis Software. Quantitative data are shown ($n = 7$, means ± SEM; two tailed Student's t test; exact P values indicated). (c) Podocin immunofluorescence. Representative confocal images of glomeruli stained for Podocin (green) and DAPI (blue). Fluorescence intensity and Podocin positive area were quantified using the same blinded imaging and analysis pipeline described above. Quantitative data are shown ($n = 7$, means ± SEM; two tailed Student's t test; exact P values indicated). Scale bars: 100 µm (immunofluorescence).

Supplementary Figure S7. Identification of apoptotic proximal tubule cells in CAZ-AVI–induced acute kidney injury using three independent proximal tubule markers.

(a) TUNEL × AQP-1 double immunofluorescence. Representative kidney sections from PBS-treated control mice (Cont) and CAZ-AVI–treated mice (1,250 mg/kg/day, subcutaneous, for three consecutive days) ($n = 7$ per group). TUNEL (red) labels apoptotic cells, and AQP-1 (green) marks proximal tubules. White arrowheads indicate apoptotic proximal tubule cells. Quantification of TUNEL-positive cells per mm^2 is shown ($P < 0.001$). Although this panel corresponds to the same experimental system as Main Figure 3b, the staining was independently repeated for revision, and the representative images differ from those shown in the main figure. (b) TUNEL × LTL double immunofluorescence. TUNEL (red) and LTL (green) demonstrate that apoptotic cells reside within LTL-positive proximal tubules. Quantification is shown ($P < 0.001$). (c) TUNEL × Megalin double immunofluorescence. TUNEL (red) and Megalin (green) confirm that apoptotic cells are Megalin-positive proximal tubule cells. Quantification is shown ($P < 0.001$). Scale bars: 100 µm (immunofluorescence). Data are presented as means ± SEM and analyzed using a

two-tailed Student's t-test. Exact P-values are indicated. Abbreviations: AQP-1, aquaporin-1; LTL, *Lotus tetragonolobus* lectin; TUNEL, terminal deoxynucleotidyl transferase–mediated digoxigenin-deoxyuridine nick-end labeling. These results collectively suggest that apoptotic cells undergoing efferocytosis originate from proximal tubules, as demonstrated by three independent proximal tubule markers.

Supplementary Figure S8. Lineage-traced proximal tubule cells undergoing apoptosis in CAZ-AVI–induced acute kidney injury. Eight-week-old SLC34a1-GCE; Rosa26-tdTomato mice were administered tamoxifen prior to CAZ-AVI exposure to induce proximal tubule–specific genetic labeling. Tamoxifen (10 mg/mL in 3% ethanol/corn oil) was injected intraperitoneally every other day for five doses, resulting in tdTomato-positive proximal tubule epithelia through activation of the eGFPCreERT2 (GCE) knock-in transgene at the endogenous *SLC34a1* locus. Following lineage labeling, mice received either PBS (control) or CAZ-AVI (1,250 mg/kg/day, subcutaneous, for three consecutive days) to induce acute kidney injury. Kidney sections were subjected to triple immunofluorescence staining for tdTomato (proximal tubule lineage marker), TUNEL (apoptotic cells), and DAPI (nuclei). Representative images show that a subset of tdTomato-labeled proximal tubule cells are TUNEL-positive, indicating that proximal tubule cells undergo apoptosis and become targets for efferocytosis in CAZ-AVI–induced injury. The apoptosis index was calculated as the ratio of TUNEL-positive cells to tdTomato-positive proximal tubule cells. An observer blinded to group allocation counted TUNEL-positive cells within tdTomato-positive tubules using fluorescence microscopy. $n = 7$ mice per group. Data are presented as means ± SEM and analyzed using a two-tailed Student's t-test. Exact P-values are indicated. Scale bars: 100 μm (immunofluorescence).

CKO vs. Cont

Efferocytosis

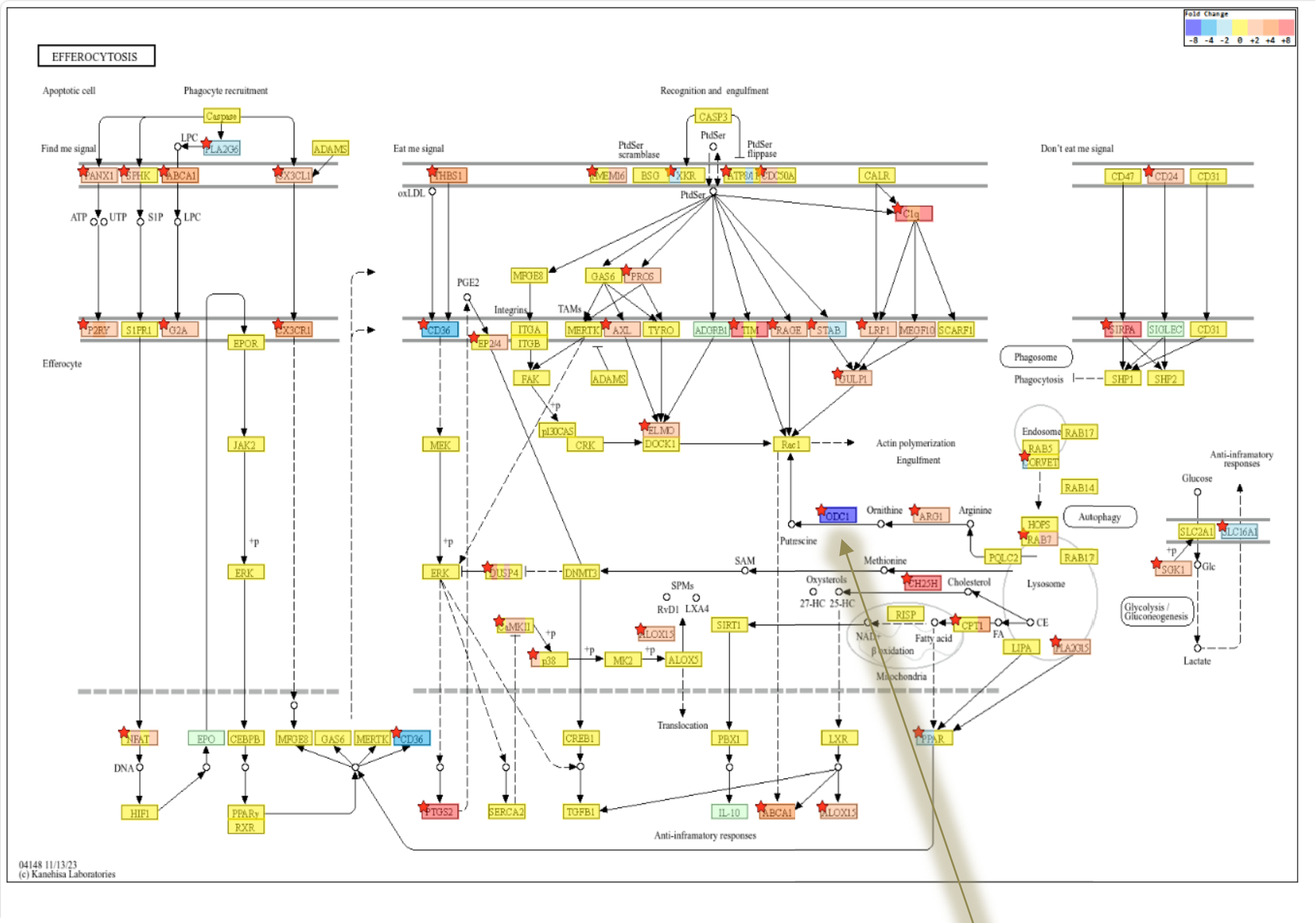

Odc1↓

Supplementary Figure 1, Hasegawa et al.

CKO vs. Cont

Glutaticone Metabolism

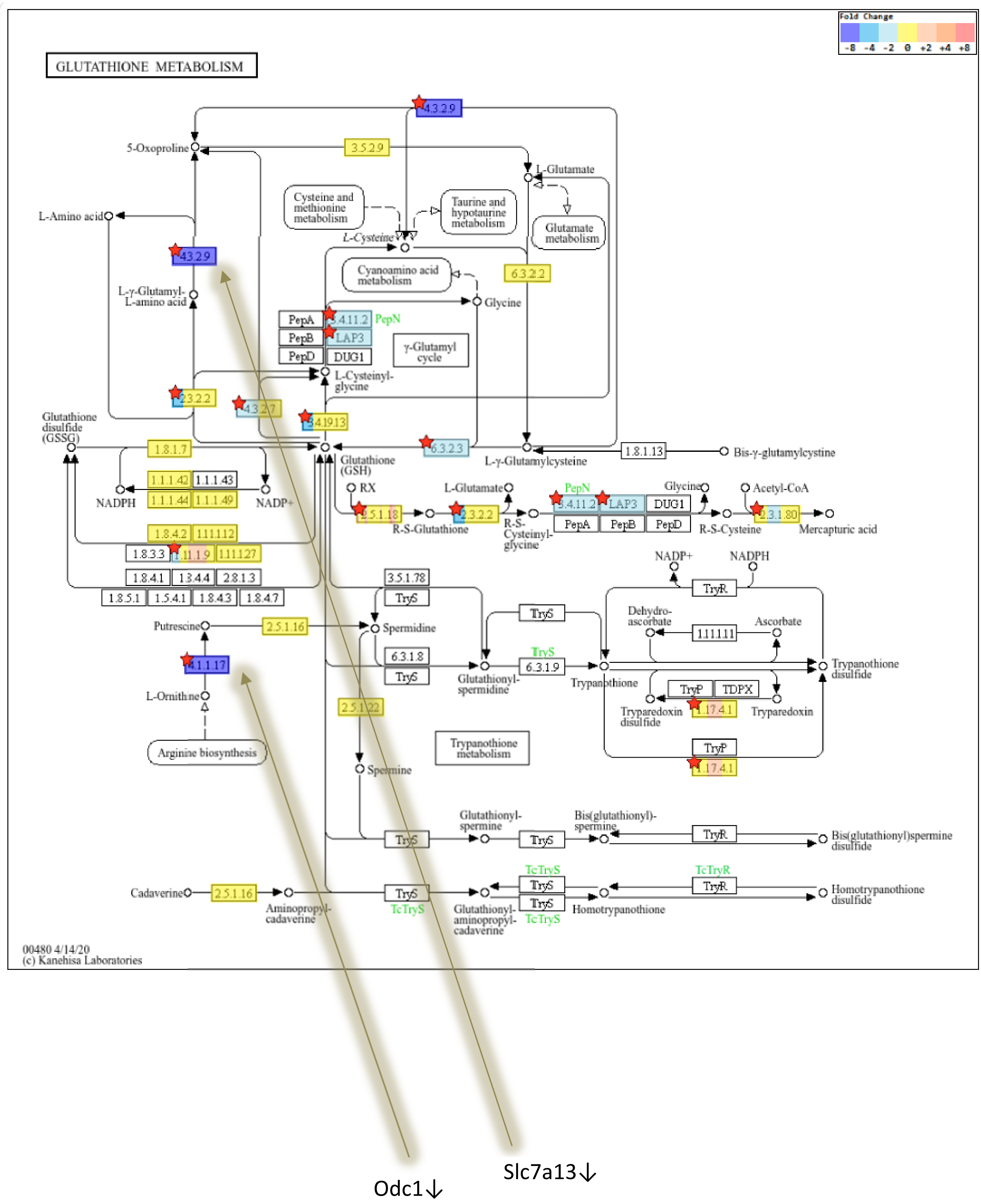

Supplementary Figure 2, Hasegawa et al.

CKO vs. Cont

Lysosome

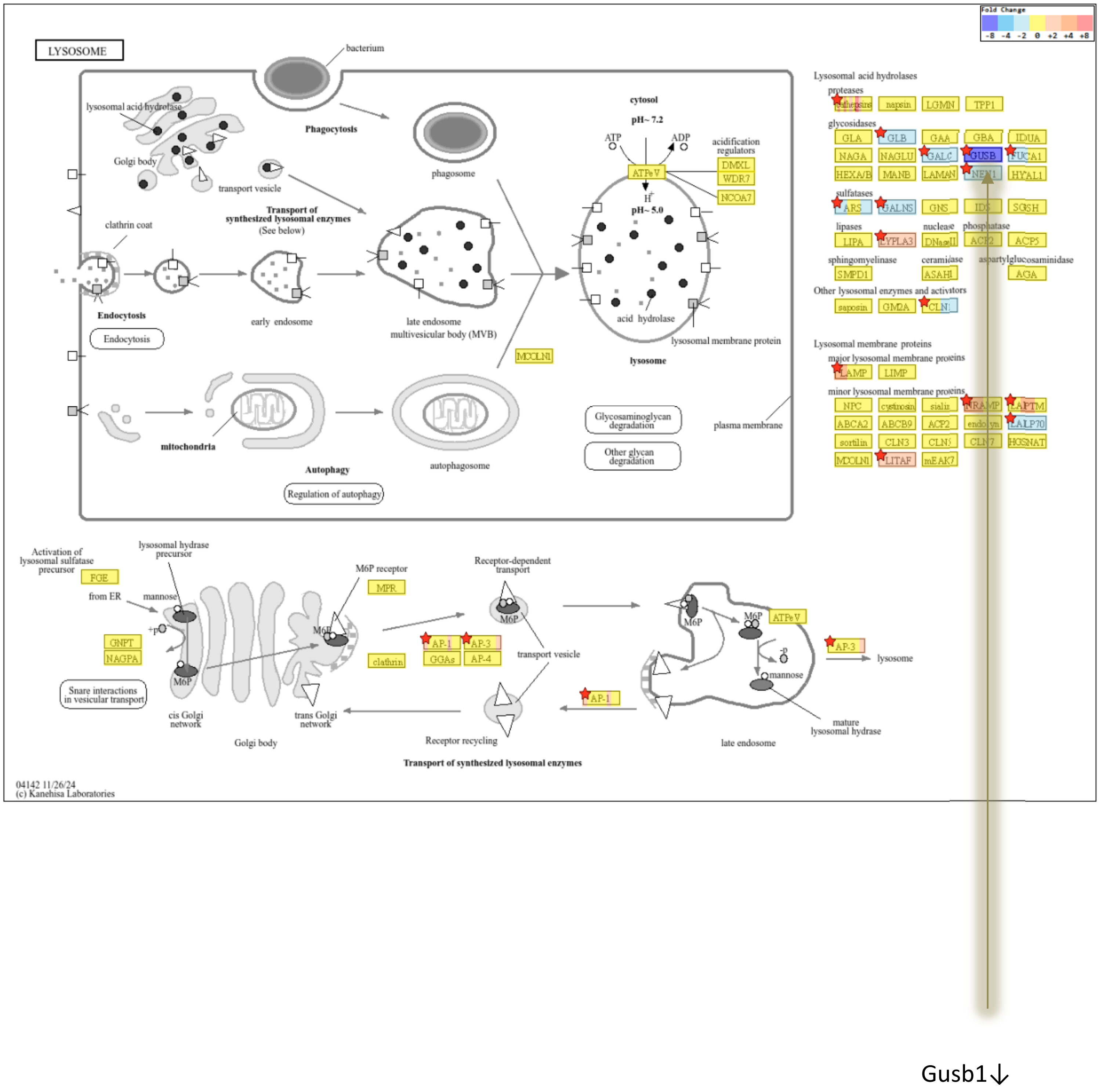

Supplementary Figure 3, Hasegawa et al.

a

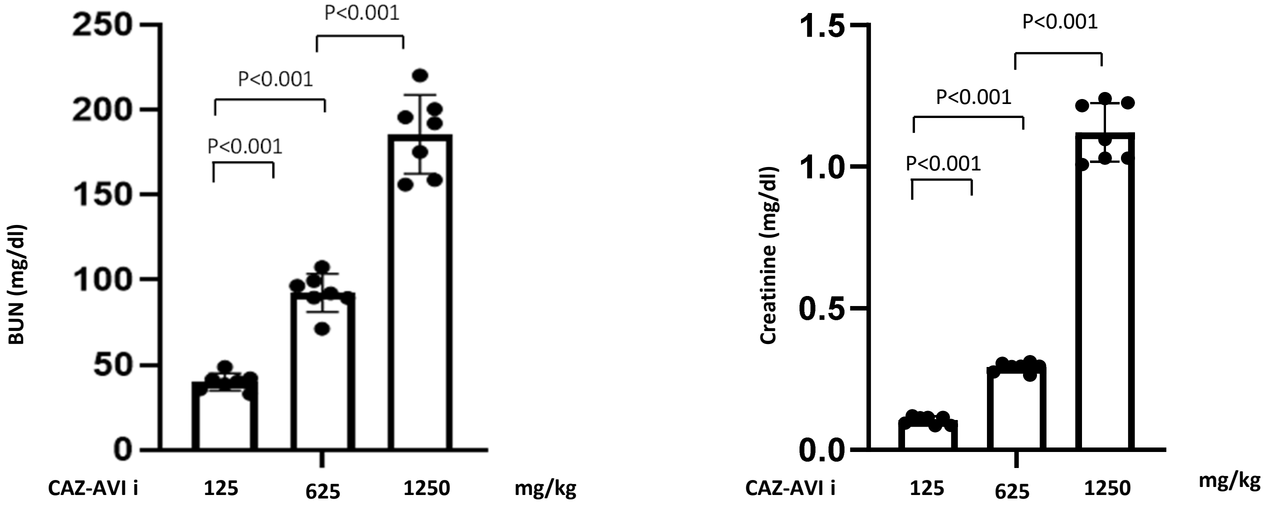

b

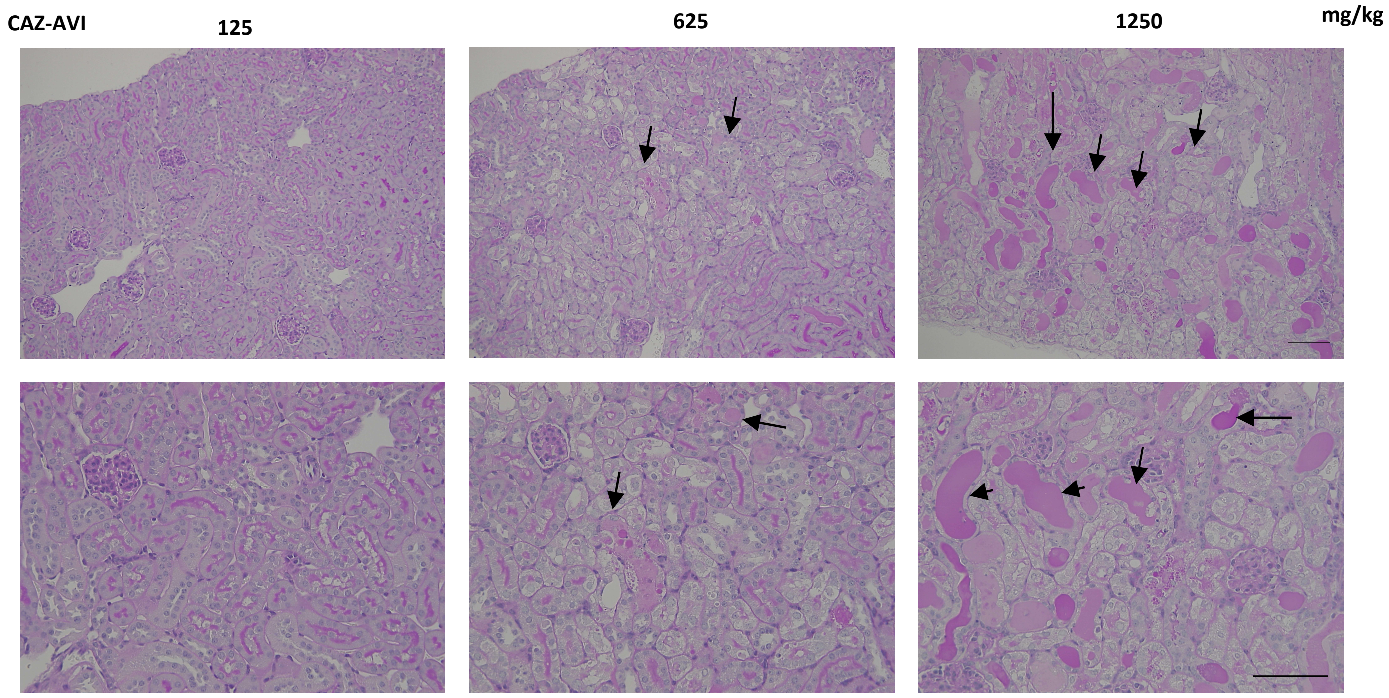

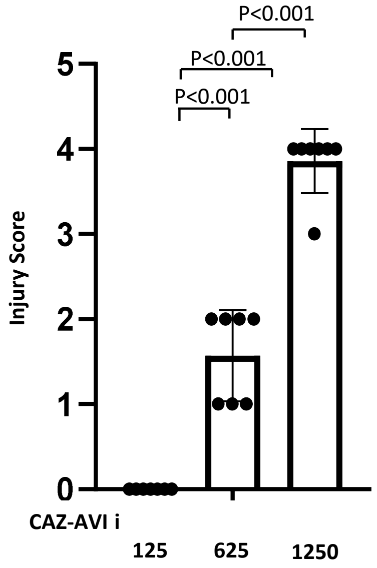

Supplementary Figure 4, Hasegawa et al.

a

Generation of Gusb flox/+
Flox PCR

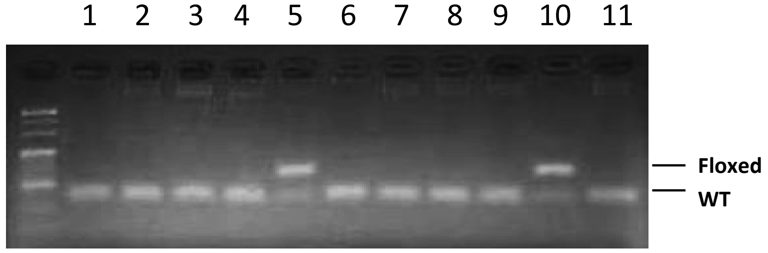

Lane	mice
1,2,3,4	+/+
2,4	fl/+

b

Validation of γ-GT Cre
Cre PCR

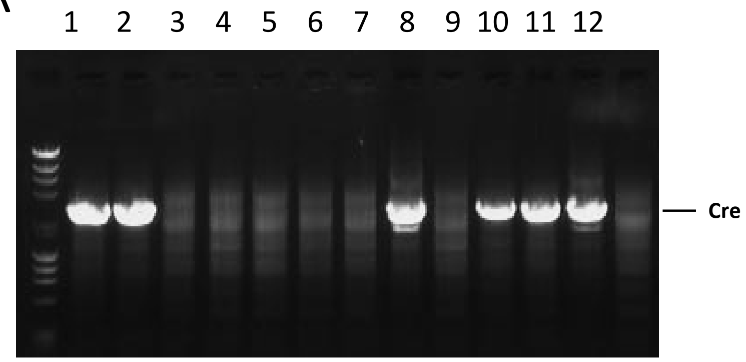

Lane	mice
3, 4, 5, 6, 7, 9	+/+
1,2 , 8, 10, 11, 12	Cre/+

c

Generation of Gusb flox/flox γ-GT Cre
= Proximal tubule-specific Gusb CAZ-AVI (1250 mg/kg s.c) mice

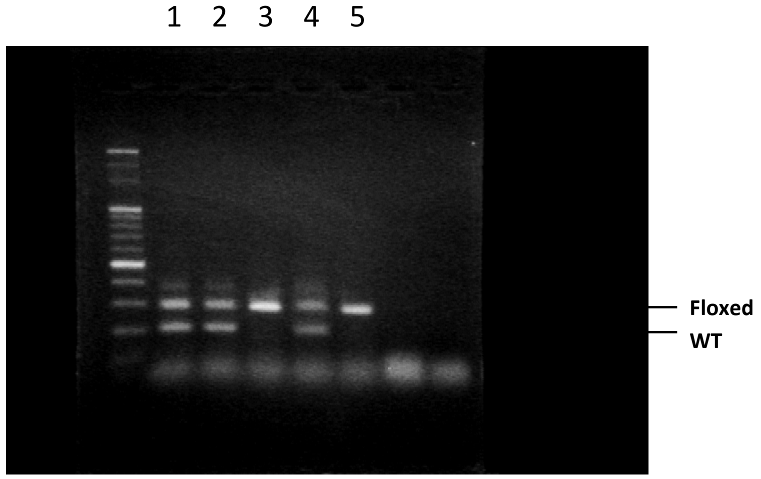

Lane	mice
3,5	fl/fl, Cre
1,2,4	fl/+ Cre

Supplementary Figure 5, Hasegawa et al.

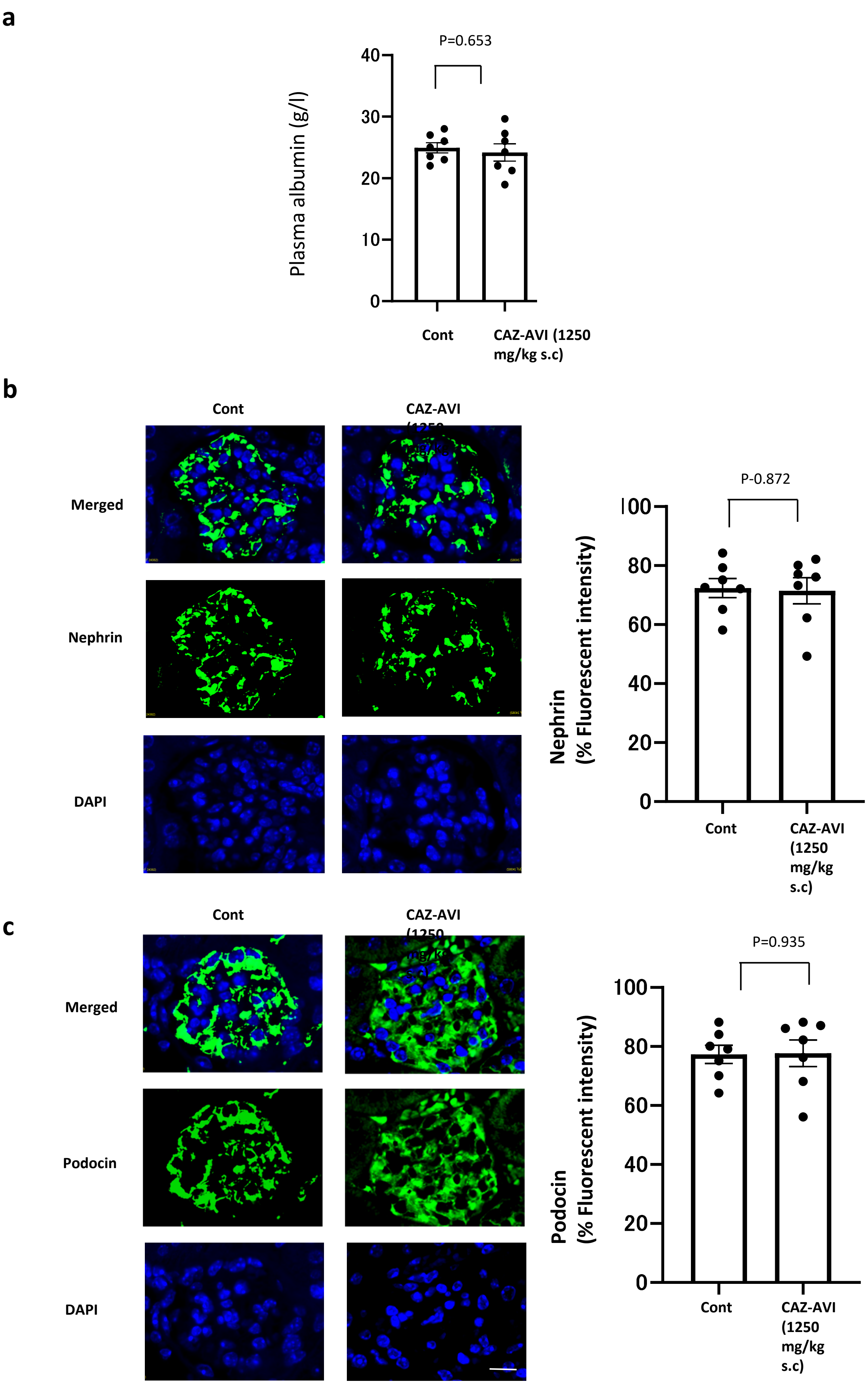

Supplementary Figure 6, Hasegawa et al.

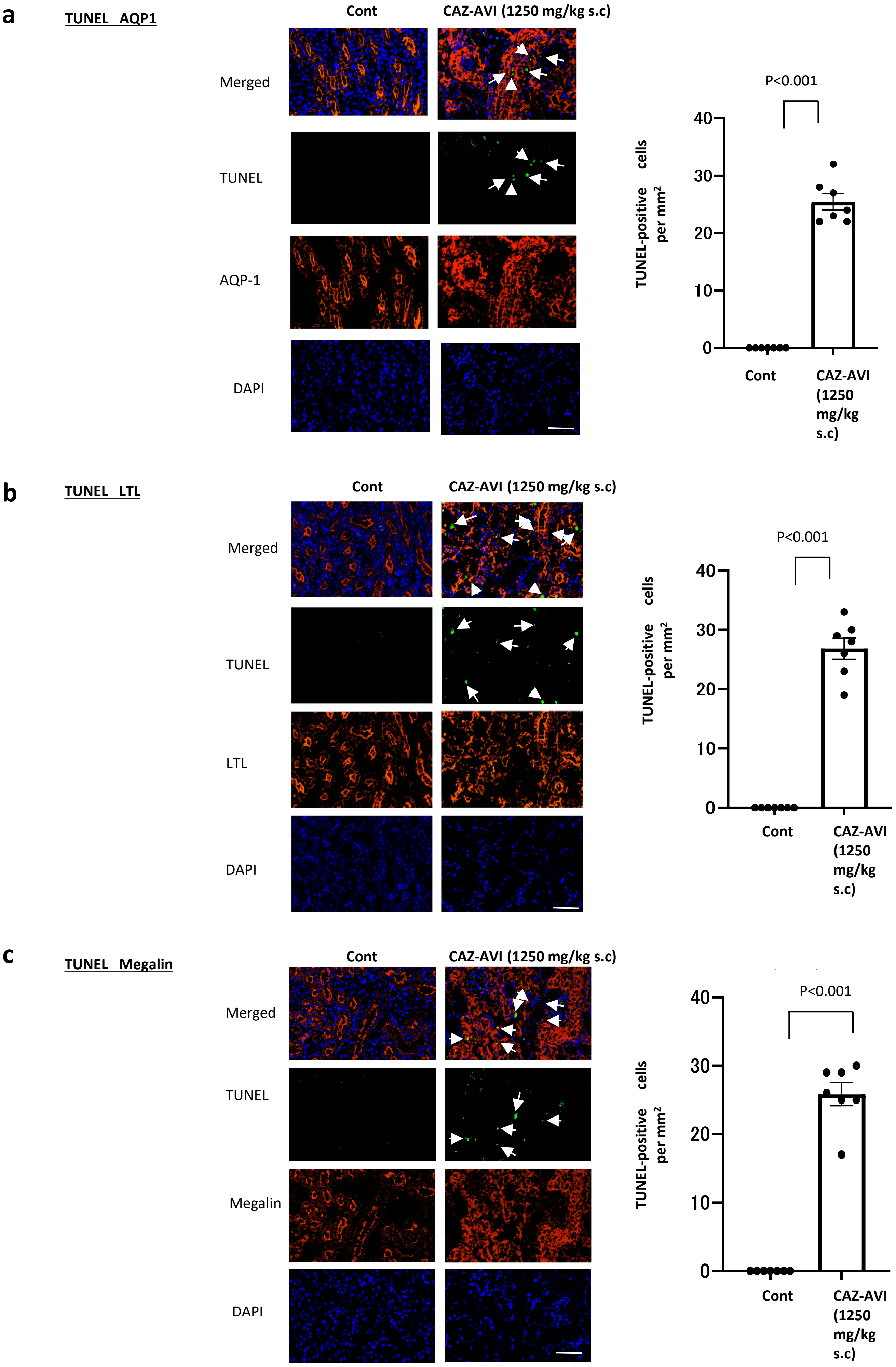

Supplementary Figure 7, Hasegawa et al.

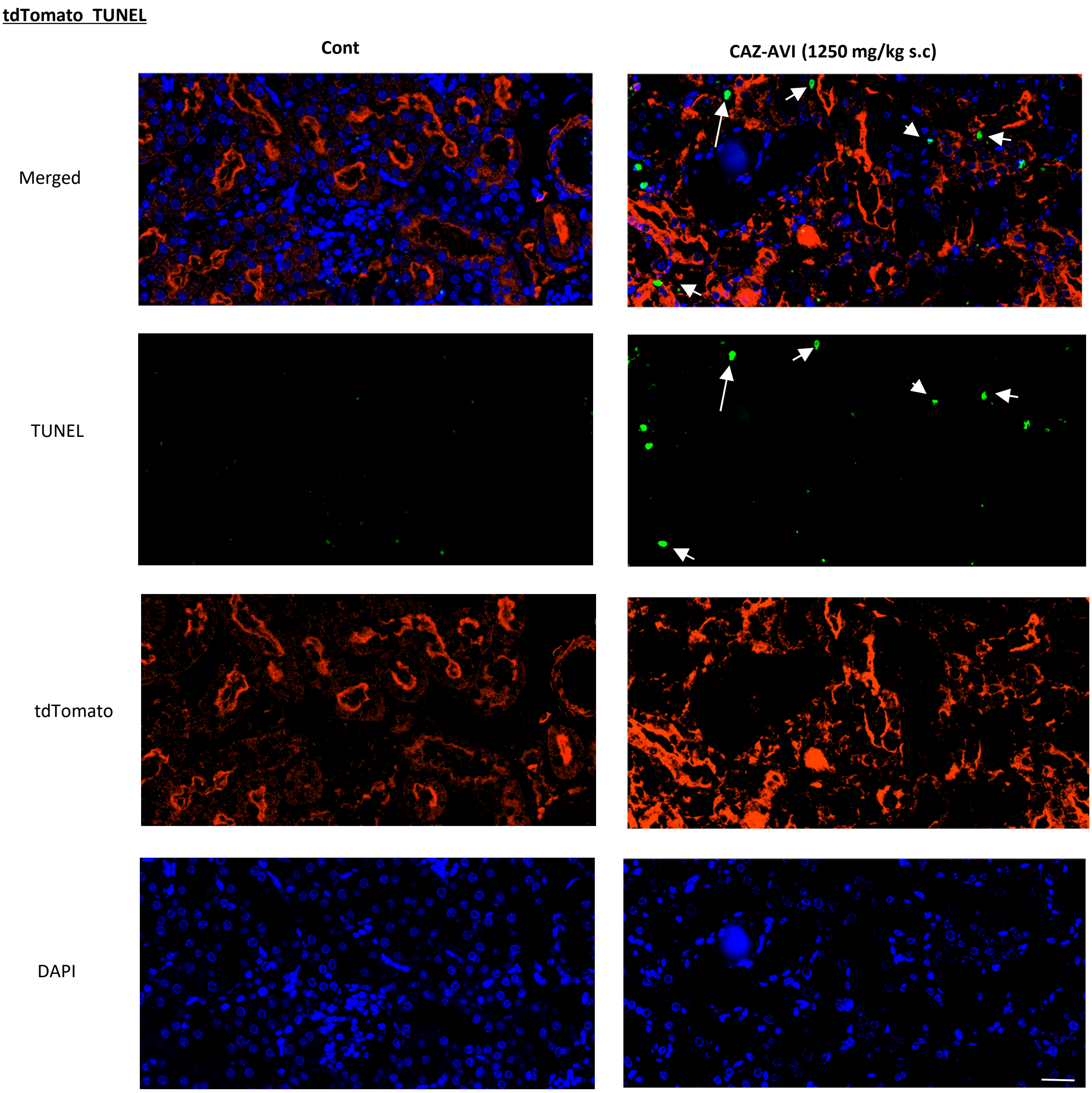

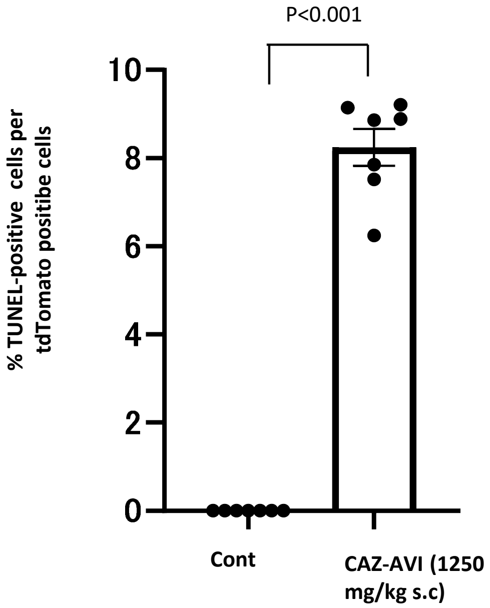

Supplementary Figure 8, Hasegawa et al.
